# A Naturally Active *Spy* Transposon Discovered from the Insect Genome of *Colletes gigas* as a Promising Novel Gene Transfer Tool

**DOI:** 10.1002/advs.202400969

**Published:** 2024-05-22

**Authors:** Mohamed Diaby, Han Wu, Bo Gao, Shasha Shi, Bingqing Wang, Saisai Wang, Yali Wang, Zherui Wu, Cai Chen, Xiaoyan Wang, Chengyi Song

**Affiliations:** ^1^ College of Animal Science & Technology Yangzhou University Yangzhou Jiangsu 225009 China; ^2^ School of Basic Medical Sciences Shenzhen University Medical School Shenzhen University Shenzhen Guangdong 518055 China

**Keywords:** chimeric antigen receptor (CAR‐T), *cgSpy*, gene delivery, transposon

## Abstract

Novel active DNA transposons, such as *Spy* transposons from the PHIS superfamily, are identified through bioinformatics in this study. The native transposases *cgSpy* and *cvSpy* displayed transposition activities of approximately 85% and 35% compared to the hyperactive *piggyBac* transposase (*hyPB*). The cgSpy transposon showed unique characteristics, including a lack of overproduction inhibition and reduced efficiency for insertion sizes between 3.1 to 8.5 kb. Integration preferences of *cgSpy* are found in genes and regulatory regions, making it suitable for genetic manipulation. Evaluation in T‐cell engineering demonstrated that *cgSpy*‐mediated chimeric antigen receptor (CAR) modification is comparable to the PB system, indicating its potential utility in cell therapy. This study unveils the promising application of the active native transposase, *Spy*, from *Colletes gigas*, as a valuable tool for genetic engineering, particularly in T‐cell manipulation.

## Graphical abstract

This study explores the discovery of novel active DNA transposons, specifically *Spy* transposons within the *PHIS* superfamily, showing recent activity across various animal species. *cgSpy* and *cvSpy* transposons from *Colletes gigas* and *Crassostrea virginica* exhibit significant transposition efficiency, with *cgSpy* demonstrating promising potential for genetic engineering applications such as T‐cell manipulation, rivaling the efficacy of the hyperactive piggyBac system.

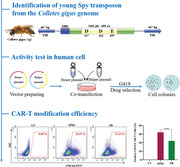

## 1 Introduction

Transposable elements (TEs) play a significant role in multicellular genomes, making up a substantial portion of an organism's genetic material. Insect genomes typically contain 15–47% transposable elements (TEs),^[^ ^1^ ^]^ whereas mammals range from 35–69%.^[^ ^2^ ^]^ Transposable elements (TEs) can be categorized into two primary categories: Class 1 (retrotransposons), which use RNA for transposition, and Class 2 (DNA transposons), employing DNA for the same purpose.^[^ ^3^, ^4^ ^]^ Cut‐and‐paste transposons, which belong to Class 2, have Terminal Inverted Repeats (TIRs) and Target Site Duplications (TSDs).^[^ ^5^ ^]^ The length and sequence of TSDs are essential for classifying cut‐and‐paste transposons into different superfamilies. For instance, the hAT superfamily commonly exhibits 8 bp TSDs,^[^ ^6^ ^]^ while the PHIS superfamily (excluding the *Spy* Family) has specific and diverse TSDs.^[^ ^7^, ^8^ ^]^ The Mutator/MuDR superfamily exhibits TSDs ranging from 9 to 12 bp.^[^ ^9^, ^10^ ^]^ RepBase identified 19 superfamilies of DNA transposons in eukaryotes based on TSD formation during transposon insertion and phylogenetic analysis of the catalytic domains (DDE/D) of transposases.^[^ ^11^, ^12^ ^]^ In addition, the catalytic domains of transposases, displaying evolutionary similarities to integrases and *RAG1* immunoglobulin gene recombinase,^[^ ^13^ ^]^ possess a crucial DDE/D motif with three negatively charged amino acids.^[^ ^14^, ^15^ ^]^ This motif plays a vital role in target site selection in the process of transposon integration.^[^ ^16^, ^17^, ^18^, ^19^, ^20^ ^]^

The transposase facilitates cut‐and‐paste transposition, which is utilized for genome manipulation and has proven useful in functional genomics, gene therapy, and transgenesis.^[^ ^21^, ^22^, ^23^, ^24^ ^]^ Cut‐and‐paste transposition holds potential applications in gene therapy, particularly in cancer immune therapy.^[^ ^25^, ^26^, ^27^ ^]^ These transposons in eukaryotes have been discovered and harnessed as genetic tools, including *Tol2*,^[^ ^28^ ^]^ *PiggyBac* (*PB*),^[^ ^29^ ^]^ *Sleeping Beauty* (*SB*),^[^ ^30^ ^]^ *Tc1*/*DD34E* transposon from Zebrafish (referred to as *ZB*),^[^ ^31^ ^]^ and *Passer* (*PS*),^[^ ^32^ ^]^ each possessing unique characteristics suitable for different purposes. *PB*, *SB*, *Tol2*, and *ZB* have demonstrated success in gene therapy, vertebrate mutagenesis, and transgenesis, but their biological aspects must be taken into consideration.^[^ ^13^, ^22^, ^31^, ^33^, ^34^ ^]^

The *Spy* transposons, a group of cut‐and‐paste transposons, have been identified in the silkworm genome.^[^ ^8^ ^]^ Along with *PIF/Harbinge*r, *ISL2EU*, *Pangu*, *NuwaI*, and *NuwaII*, *Spy* transposons are evolutionarily related and classified within the same superfamily known as “*PHIS*”.^[^ ^8^, ^35^ ^]^ This superfamily exhibits high polymorphism in target sequences, coding capacity, and conserved motifs of the transposase.^[^ ^8^, ^35^ ^]^ Additionally, *Spy* transposons differ from most known DNA transposons in their strong preference for insertion within the AAATTT motif and the absence of TSDs upon insertion.^[^ ^8^, ^35^ ^]^ These unique characteristics may offer distinct potential applications in gene therapy and transgenesis when compared to well‐established transposons such as *PS*, *ZB*, *SB*, *PB*, and *Tol2*.^[^ ^13^, ^22^, ^31^, ^32^, ^33^, ^36^ ^]^

Our objective was to broaden the application of genetic tools in the life sciences by investigating the distribution of *Spy* transposons and identifying active members within this family. We assessed the activity of *Spy* transposons derived from five species – *Colletes gigas*, *Crassostrea virginica*, *Hedya salicella*, *Cerceris rybyensis*, and *Lymantria dispar* – in human cells. Through our experiments, we discovered a highly active *Spy* transposon isolated from *Colletes gigas*, which we designated as *cgSpy*. Subsequently, we conducted a comprehensive analysis to examine its capacity to carry genetic cargo and its preferences for integration into the human genome on a genome‐wide scale.

## 2 Results

### 2.1 Recent and Current Activities of *Spy* Transposons in Multiple Invertebrate Lineages

**Figure 1:**
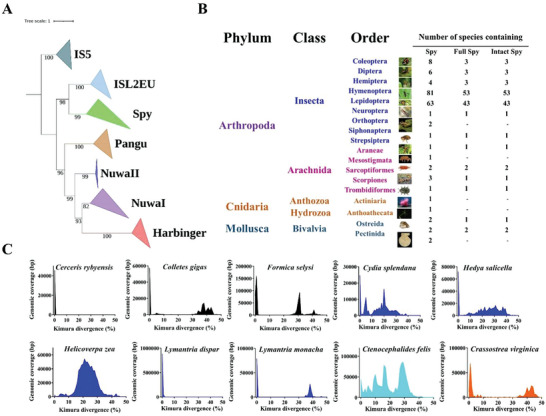
Phylogeny, taxonomy, and evolutionary dynamics of *Spy* transposons. A) A phylogenetic tree of *Spy* transposases is shown, along with other members of the PHIS superfamily. The tree was constructed using the maximum likelihood method in IQ‐TREE, with bootstrap support values. B) The distribution of *Spy* transposons across three phyla is shown: Arthropods, Cnidaria, and Mollusca. The number beside each species picture indicates the number of *Spy* transposons detected in that phylum. Full‐length (FL) *Spy* refers to *Spy* transposons with two detectable TIRs, while intact *Spy* refers to full‐length *Spy* transposons that encode intact transposases (>500 amino acids, containing DNA‐binding domain and DDE domain). C) The K‐divergence of *Spy* transposons was investigated across ten genomes. Using RepeatMasker utility scripts, we calculated the K‐divergence based on either the consensus sequence or the representative sequence for each transposon. The y‐axis represents the coverage (in base pairs, bp) of each *Spy* transposon within the genome, while the x‐axis indicates the K‐divergence (%). Black, blue, and sky‐blue points represent species from three different Arthropod orders: Hymenoptera, Lepidoptera, and Siphonaptera, respectively. Species represented by orange points belong to the Ostreida order of the class Bivalvia (Mollusca).

A comprehensive analysis of *Spy* elements was conducted to redetermine their distribution across species using the TBlastN search method as described in the methods section. Overall, we identified 182 *Spy* transposons in 172 species (Table S1, Supporting Information), including those (54 *Spy* transposons in 21 species) previously reported. The phylogenetic analysis confirmed that all obtained elements belong to the *Spy* family and transposases from *Spy* and representative transposases from *NuwaI*, *NuwaII*, *ISL2EU*, *Pangu*, *PIF/Harbinger*, and *IS5* (as the outgroup) form six separate and well‐supported monophyletic clades. Notably, *ISL2EU* and *Spy* are found to be sister clades in the analysis (**Figure** **1**A; Figure S1, Supporting Information). Our findings also revealed that *Spy* transposons are exclusively found in invertebrates (Figure 1B), mainly distributed in the phylum of Arthropoda, including nine orders (167 transposons in 159 species) of insects and five orders (8 *Spy* transposons in 7 species) of Arachnida, but very few in Cnidaria (3 *Spy* transposons in 2 species) and Mollusca (4 *Spy* transposons in 4 species). The intact *Spy* transposons vary from 1165 bp to 5910 bp in length and have one open reading frame. The TIRs of different *Spy* transposons vary greatly in length (ranging from 5 to 691 bp), and typical insertion preference sequence motifs in the 5′ flanks (5′‐AAA‐3′) and 3′ flanks (5′‐TTT‐3′) were detected for most *Spy* transposons in genomes (Table S1, Supporting Information).

We identified 116 intact *Spy* transposons, designated *Spy*, encoding transposases with over 500 amino acids that include a DNA‐binding domain and a DDE motif, and flanked by terminal inverted repeats (TIRs). These elements were found in 108 species spanning multiple animal phyla. (Figure 1B; Table S1, Supporting Information). This suggests that they may have invaded these lineages recently and remain active in some genomes. We selected ten *Spy* transposons with high intact copy numbers (>5) in the genomes to evaluate their activity using K divergence as described in the Methods section. The data analysis revealed that most *Spy* copies in some genomes, such as *Cerceris rybyensis*, *Colletes gigas*, *Formica selysi*, *Lymantria dispar*, *Lymantria monacha*, *Hedya salicella*, and *Crassostrea virginica*, represent very low K divergences (close to zero), indicating activity dominated by young *Spy* transposons (Figure 1C). In contrast, *Spy* transposons in other genomes (*Cydia splendana*, *Helicoverpa zea*, *Chymomyza costata*, *Ctenocephalides felis*) are represented by multiple invasion waves, indicating the coexistence of young and old *Spy* copies in these genomes (Figure 1C).

### 2.2 Natively Active *Spy* Transposon from Insect Genomes

**Figure 2:**
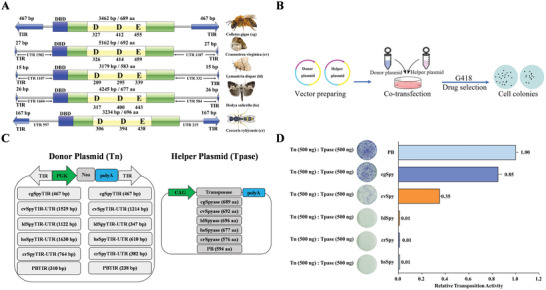
Transposition Activities of *Spy* Elements in Human Cells. A) The structural and functional components of representative *Spy* transposons from *Colletes gigas*, *Crassostrea virginica*, *Hedya salicella Cerceris rybyensis*, and *Lymantria dispar* are depicted. The elements contain a single gene that encodes the transposase (green rectangle). The blue arrows represent TIRs, the blue rectangle represents the DNA‐binding domain (DBD), and the yellow rectangle represents the catalytic domain (DDE). B) Binary transposition system includes simultaneous transfection of a donor transposon and a helper plasmid encoding transposase. Donor plasmid has a gene cassette for promoter activity and antibiotic resistance flanked by TIRs, conferring resistance upon chromosomal transposition. Helper plasmid contains a transposase expression cassette controlled by the regulatory promoter. In cell‐based assay, vectors co‐transfected into cells and subjected to drug selection. Active transposase increases resistant colony formation compared to controls without transposase. C) Donor and helper plasmids used in human cells. Donor plasmids: the white arrows represent transposon TIRs including cg*Spy*TIR, cv*Spy*TIR, hs*Spy*TIR, cr*Spy*TIR, ld*Spy*TIR, and *PB*TIR, the donor vectors of cv*Spy*TIR, hs*Spy*TIR, cr*Spy*TIR, and ld*Spy*TIR also contain 5′ and 3′ UTRs of transposases; PGK, PGK promoter; Neo, neomycin resistance gene. Helper plasmids: CAG, CAG promoter; transposase, the transposase (cg*Spy*ase, cv*Spy*ase, hs*Spy*ase, cr*Spy*ase, ld*Spy*ase, and *hyPB*ase) open reading frame (ORF). D) Transposition activities of five *Spy* transposases (cg*Spy*, cv*Spy*, hs*Spy*, cr*Spy*, ld*Spy*) were evaluated in HeLa cells co‐transfected with high amounts of transposon DNA (500 ng). Three replicates were performed for each group. The dishes represent G418‐resistant cell colonies stained by Giemsa.

According to the analysis of their evolutionary dynamics and the abundance of intact copies in genomes, *Spy* transposons appear to have recently invaded the genomes of certain invertebrate species and may exhibit high activity levels. Specifically, *Spy* transposons found in *Colletes gigas*, *Crassostrea virginica*, *Hedya salicella*, *Cerceris rybyensis*, and *Lymantria dispar* are recent invasions characterized by high transposase and TIR identities (>97%, Table S2, Supporting Information) and minimal K divergences (**Figure** **2**A), suggesting their potential for transposition activity. These transposons were chosen for activity evaluation in human cells and were designated as cg*Spy*, cv*Spy*, hs*Spy*, cr*Spy*, and ld*Spy* respectively. These transposons share a similar structural organization, comprising a solitary ORF that encodes a transposase consisting of ≈700 amino acids. The transposase is flanked by TIRs of differing lengths (467, 167, 27, 26, and 15 bp), respectively (Figure 2A; Table S2, Supporting Information).

To investigate the transposition capabilities of *Spy* within diverse genomes, a binary transposition system was utilized, as depicted in Figure 2B. This method entails the simultaneous transfection of a donor transposon and a helper plasmid encoding transposase.^[^ ^31^, ^32^ ^]^ The donor plasmid comprises a gene cassette for promoter activity and antibiotic resistance, delineated by terminal inverted repeats (TIRs), which can impart drug resistance to cells when transposed into their chromosomes. Conversely, the helper plasmid contains a transposase expression cassette under the control of a regulatory promoter element. Subsequently, these engineered vectors undergo evaluation through a cell‐based assay, leveraging a dual plasmid‐based transposition approach. Here, the donor plasmid is amalgamated with the helper plasmid and co‐transfected into cultured cells, followed by a drug selection phase. An active transposase that exhibits substantial transposition activity within the cells will induce a notably higher count of resistant colonies when contrasted with the control (cells devoid of any source of transposase).

We designed five transposon donor vectors, each containing the same Neo expression cassette and backbone. However, each vector possessed a distinct TIR sequence. One type of TIR, derived from the genome of *Colletes gigas* (cg*Spy*TIR), was long, measuring 467 bp. Conversely, the four other types, from *Cerceris rybyensis* (cr*Spy*TIR), *Crassostrea virginica* (cv*Spy*TIR), *Hedya salicella* (hs*Spy*TIR), and *Lymantria dispar* (cr*Spy*TIR) genomes, were short, measuring 167, 26, 27, and 15 bp, respectively. The untranslated regions (UTRs), the sequences between the TIR and the ORF, were also included for the transposon donor vectors (cv*Spy*TIR, hs*Spy*TIR, cr*Spy*TIR, and ld*Spy*TIR) with short TIRs, as shown in Figure 2C. Additionally, we synthesized five helper plasmids containing transposase‐coding sequences obtained from the genomes of *Colletes gigas*, *Crassostrea virginica*, *Hedya salicella*, *Cerceris rybyensis*, and *Lymantria dispar*. We subsequently subcloned the verified coding sequence clones into their corresponding expression vectors: pCAG‐*cgSpy*, pCAG‐*cvSpy*, and pCAG‐hs*Spy*, pCAG‐*crSpy*, and pCAG‐*ldSpy* as shown in Figure 2C.

The results of the transposition assay showed that the transposases *cgSpy* and *cvSpy*, derived from the genomes of *Colletes gigas* and *Crassostrea virginica*, respectively, exhibited significant transposition activity. In contrast, none of the transposase clones (*hsSpy*, *crSpy*, and *ldSpy*) derived from *Hedya salicella*, *Cerceris rybyensis*, and *Lymantria dispar*, respectively, exhibited detectable transposition activity (Figure 2D). Furthermore, the transposon donor plasmids of cv*Spy*, hs*Spy*, cr*Spy*, and ld*Spy* with the short TIRs were not transpositionally functional, as shown in Figure S1A–C (Supporting Information). Overall, *cgSpy* and *cvSpy* displayed transposition activities of ≈85% and 35%, respectively, compared to the hyperactive *piggyBac* (hy*PB*) transposase^[^ ^37^ ^]^ (Figure 2D). The *cgSpy* system consisted of two crucial components: a donor plasmid, pcg*Spy*‐PGK‐Neo, and a helper plasmid, pCAG‐*cgSpy*, which were used in subsequent experiments.

### 2.3 Optimization of *cgSpy* TIRs

**Figure 3:**
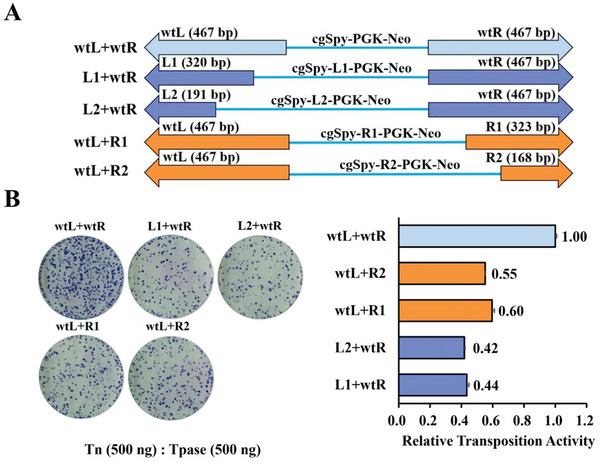
Transposition activities of the native *cgSpy* transposon and its optimized terminal inverted repeats in human cells. A) Structural organization of the *cgSpy* transposon and its optimized TIR vectors, including wild‐type left and right TIRs (wtL+wtR), L1+wtR, L2+wtR, wtL+R1, and wtL+R2. B) Transposition activities of the *cgSpy* transposase in HeLa cells co‐transfected with 500 ng of each transposon DNA (wtL+wtR (*cgSpy*), L1+wtR, L2+wtR, wtL+R1, and wtL+R2). Three replicates were performed for each group. The dishes represent G418‐resistant cell colonies stained with Giemsa.

To define the necessary TIR sequences of *cgSpy* for efficient transposition, four *cgSpy* transposons TIR variants (L1+wtR, L2+wtR, wtL+R1, wtL+R2) with different lengths were successfully constructed (shown in **Figure** **3**A) using standard molecular cloning techniques and confirmed by Sanger sequencing. Transposition activity in HeLa cells revealed that wild‐type *cgSpy* TIRs had the highest activity, while all TIR variants of *cgSpy* showed reduced activity: wtL+R1 at 60%, wtL+R2 at 55%, L1+wtR at 44%, and L2+wtR at 42% of wtL+wtR (*cgSpy*) activity (Figure 3A,B).

### 2.4 No Typical Overproduction Inhibition of *cgSpy* Transposon System

In this study, we investigated the putative overproduction inhibition (OPI) of the *cgSpy* transposon system, which occurs due to excessive transposase expression hindering transposition activity. This phenomenon has been observed for the most well‐defined transposons.^[^ ^31^, ^32^, ^36^, ^38^ ^]^ For comparison, we utilized the *SB* (SB100X transposase) and *PB* (hy*PB* transposase) transposon systems in parallel.^[^ ^37^, ^39^ ^]^ We employed donor and helper plasmids with identical backbones to minimize discrepancies among the cg*Spy*, *SB*, and *PB* systems. The only variable components were the transposon‐specific TIRs and the transposase‐coding sequences, which varied based on the transposons utilized.

**Figure 4:**
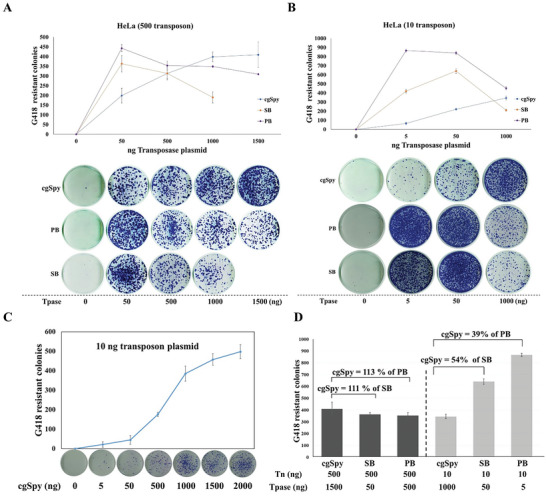
Transposition efficiency of *cgSpy*, SB, and *PB* transposons in human cells. A,B) Comparative transposition activities of *cgSpy*, SB, and *PB* transposons in HeLa cells co‐transfected with varying amounts of transposon DNA (10 and 500 ng). Each group was analyzed in triplicate. C) Transposition activities in HeLa cells co‐transfected with *cgSpy* transposons under different transposon DNA conditions. Slight increases in transposition were observed from 1000 to 1500 ng and from 1500 to 2000 ng, indicating that saturation may not have been reached even at high transposon DNA concentrations (1000 to 2000 ng). D) Comparative transposition activities of *cgSpy* and *SB* transposons in HeLa cells under high and low transposon DNA conditions. Error bars represent the standard deviation of three independent replicates.

The OPI was evaluated at low (10 ng) and high (500 ng) dosages of transposon donor plasmids. The findings demonstrated that *cgSpy* did not exhibit any OPI at either dosage (**Figure** **4**). The transposition activity of *cgSpy* increased proportionally to the level of transfected plasmids, reaching a plateau at 1000 ng for the high dosage (Figure 4A). However, at the low dosage, its activity increased mildly from 50 to 1000 ng compared to *SB* and *PB* in the parallel experiment (Figure 4B). Furthermore, it increased slightly from 1000 to 1500 ng, and from 1500 to 2000 ng. Notably, it did not reach a plateau even at very high amounts (1000 to 2000 ng) of transposase plasmids (Figure 4C). In contrast, both *SB* and *PB* demonstrated OPI at both low and high dosages, albeit with variations in peak activity. For the high dosage, both *SB* and *PB* reached peak activity at 50 ng of transfected transposase plasmid, while at the low dosage, *PB* achieved peak activity at 5 ng, compared to *SB* which required 50 ng. It is noteworthy that *SB* and *PB* did not exhibit a plateau effect after reaching their activity peaks but experienced a decrease in their activity, consistent with the OPI phenomenon. Conversely, *cgSpy* did not demonstrate the same pattern as *SB* or *PB* (Figure 4A,B). Additionally, *cgSpy* exhibited transposition activity comparable to *SB* (111%) and *PB* (113%) at high dosage (Figure 4D), but lower than that of *SB* (54%) and *PB* (39%) at low dosage (Figure 4E). Overall, our observations indicate that *cgSpy* demonstrates high transposition activity in mammalian cells, without displaying the typical OPI phenomenon during transposition.

### 2.5 Cargo Capacity and Insertion Copy Number of *cgSpy* Transposon

**Figure 5:**
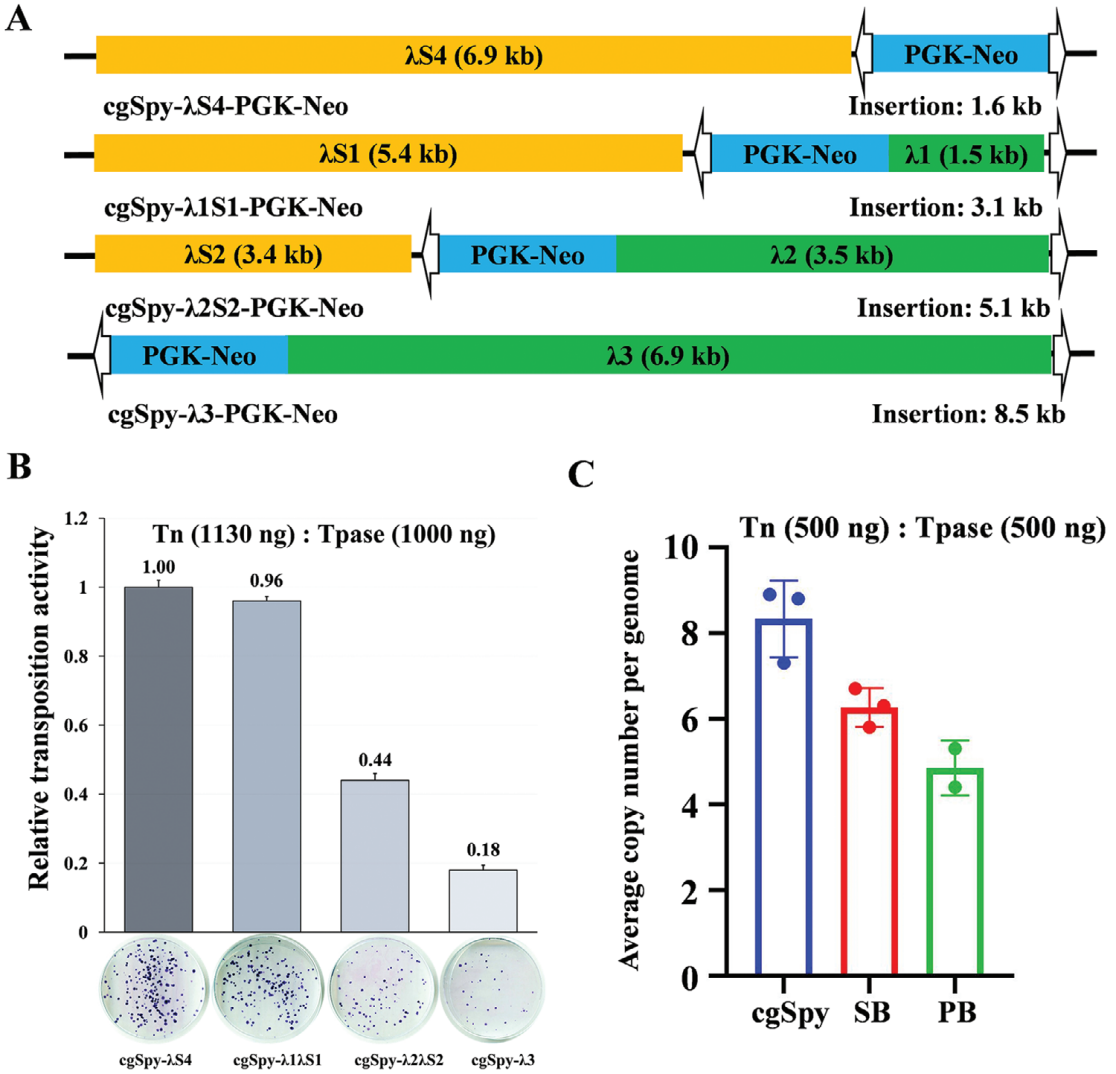
Cargo capacity of *cgSpy* in human cells. A) Different‐sized fragments are used as donor plasmids in human cells. The *cgSpy*TIRs are represented by the white arrows, while PGK signifies the PGK promoter and Neo indicates the neomycin resistance gene. An inner DNA fragment, λ kb (λ 1, λ 2, λ 3 = 1.5, 3.5, and 6.9 kb), and an outer stuffer fragment, λ S kb (λ S1, λ S2, λ S4 = 5.4, 3.4 and 6.9 kb), are used. B) The transposition activity decreases as the insertion size increases, as observed from the data in panel A. Three replicates were performed for each group, and the G418‐resistant cell colonies stained by Giemsa are displayed in the dishes below. C) Represents the insertion copy number of *cgSpy*. The average number of Neo gene insertions per Hela cell genome, mediated by cg*Spy*, SB, and *PB* transposon systems, was measured using two independent digital droplet PCR assays. The vectors used in this assay were of equal size.

Four donor plasmids were co‐transfected with the helper plasmid into HeLa cells to test the cargo capacity of the *cgSpy* system. These plasmids contained inserted fragments of varying sizes, ranging from 1.6 to 8.5 kb, placed between the TIRs (**Figure** **5**A). The results showed a decrease in the efficiency of transposition by the *cgSpy* system as the size of the inserted fragments increased. The donor plasmid with the shortest fragment (1.6 kb) exhibited the highest transposition activity, whereas the donor plasmid with the longest fragment (8.5 kb) had the lowest colony numbers (Figure 5B). Notably, the decrease in transposition activity is minimal (only 4%) when the insertion size increases from 1.6 to 3.1 kb. However, a significant drop in transposition activity (≈50%) was observed when the insertion sizes increased from 3.1 to 5.1 kb or from 5.1 to 8.5 kb. Furthermore, the transposition activity of the *cgSpy* system decreased by ≈80% when the insertion size increased from 1.6 to 8.5 kb (Figure 5B). On average, the transposition activity of the *cgSpy* system decreased by ≈12% with each kilobase (kb) increase in insertion size. In contrast, previous reports indicate that the efficiency of the *SB* system decreased by ≈30% per kb increase,^[^ ^40^ ^]^ while *PS* exhibited a decrease of ≈12% per kb increase in insertion size.^[^ ^32^ ^]^ The insertion copy number of *cgSpy* was about eight per HeLa cell genome, which is slightly higher than that measured for *SB* (≈6) and *PB* (≈5) in HeLa cells (Figure 5C).

### 2.6 Genome‐Wide Insertion Profile of *cgSpy* in Human HepG2 Cell

**Figure 6:**
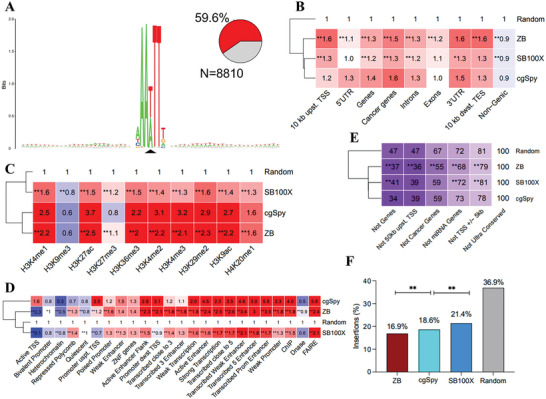
A) Integration sites of the *cgSpy*. The sequence logos show the majority‐rule consensus sequences at the genomic insertion loci in a 60‐bp window, centered around the target AAATTT (*cgSpy*) nucleotides. The value 2 (log2 4) on the y‐axis stands for the maximum possible frequency. The pie charts depict the percentages of insertions occurring at the transposon‐specific AAATTT (*cgSpy*) consensus motif. The N values represent the numbers of uniquely mappable transposon insertions. B) Integration loci of *cgSpy*, SB100X, and *ZB* were counted in gene‐related segments of the human genome. The numbers represent fold change increase (red) or decrease (blue) in insertion frequencies compared to a theoretical random control (set to 1). C) Relative co‐occurrence of transposon integration sites with histone tail modifications. The numbers represent fold change increase (red) or decrease (blue) in insertion frequencies within ChiP‐Seq peaks compared to a theoretical random control (set to 1). The dendrogram on the left is based on the mean frequency values of the rows. D) Insertion site frequencies in functional genomic categories. The categories on the *x*‐axis were established using combined histone modification based on ChIP‐Seq, RNA‐Seq, and DNase‐Seq datasets for the HepG2 cell line. The numbers in the boxes are fold change values above the random expected frequencies (arbitrarily set to 1). The dendrogram on the left is based on row means. The red and blue colors stand for over‐ and under‐representation, respectively. E,F) Insertion frequencies of *ZB*, *cgSpy*, and *SB100X* in genomic safe harbors. Statistical significance (Fisher's exact test) measured for the values of the *ZB* and *SB100X* versus *cgSpy* is indicated by stars; ^*^ *p* < 0.05, ^**^ *p* < 0.001.

To gain a deeper understanding of the genome‐wide insertion preferences of *cgSpy* transposons, we performed high‐throughput sequencing to profile their integration sites in the HepG2 cell genome. We also compared these integration sites to those of *SB* and *ZB* transposons, which are known to exhibit relatively random insertion patterns throughout the genome.^[^ ^31^ ^]^ In total, we obtained 8810 unique integration sites of *cgSpy* throughout the genome using the annotation protocol described in the Methods section. The majority (≈60%) of *cgSpy* transposons were observed to be inserted into specific target sites consisting of the 5′‐AAWTT‐3′ sequence. These target sites are commonly surrounded by regions rich in AT‐rich DNA (**Figure** **6**A). Our analysis of the alignments of the genomic flanks of *Spy* transposon insertions in the human genome with their respective original genomic loci revealed that *Spy* does not generate target site duplications (TSDs), as shown in Figure S2 (Supporting Information).

In general, *cgSpy* exhibits a slight preference for integrating into genes, while demonstrating a stronger preference for transcription‐promoting regulatory regions. Our data analysis further reveals that *cgSpy* shows a slight preference for inserting into the 5′ ends of genes, particularly genes and cancer genes in HepG2 cells. Moreover, we observed similar or lower frequencies of integration toward the upstream of TSSs and downstream of TESs, compared to *ZB* and *SB* transposons (Figure 6B). Further analysis reveals that *cgSpy* exhibits a strong preference for inserting into genomic segments that possess specific histone marks associated with transcription and activating gene regulatory regions. Additionally, *cgSpy* displays high insertion frequencies in open chromatin regions and promoter and/or enhancer regions, particularly when these chromatin segments are related to active gene expression and the activation of specific histone marks near the 5′ end of transcripts. These patterns were observed in comparison to *ZB* and *SB* transposons (Figure 6C,D).

We analyzed the insertion site datasets of *cgSpy* with the frequencies of integration into genomic safe harbors (GSHs), which are specific regions of the human genome that can accommodate newly integrated DNA without causing any harm to the host cell or organism. The values of *cgSpy* insertions, outside of cancer genes, miRNA genes, and regions within 50 kb upstream of TSSs, are comparable to those of *SB* and higher than those of ZB. In contrast, *cgSpy* insertions occur outside of genes and the regions of TSS +/‐ 5 kb have slightly lower values (34% and 78%) compared to those of *ZB* and *SB* (Figure 6E). By simultaneously applying all five GSH criteria, we found that 21.4% of *SB* insertions, 18.6% of *cgSpy* insertions, and 16.9% of *ZB* insertions occur within a GSH (Figure 6F).

### 2.7 Efficient Gene Delivery in Human T Cells

**Figure 7:**
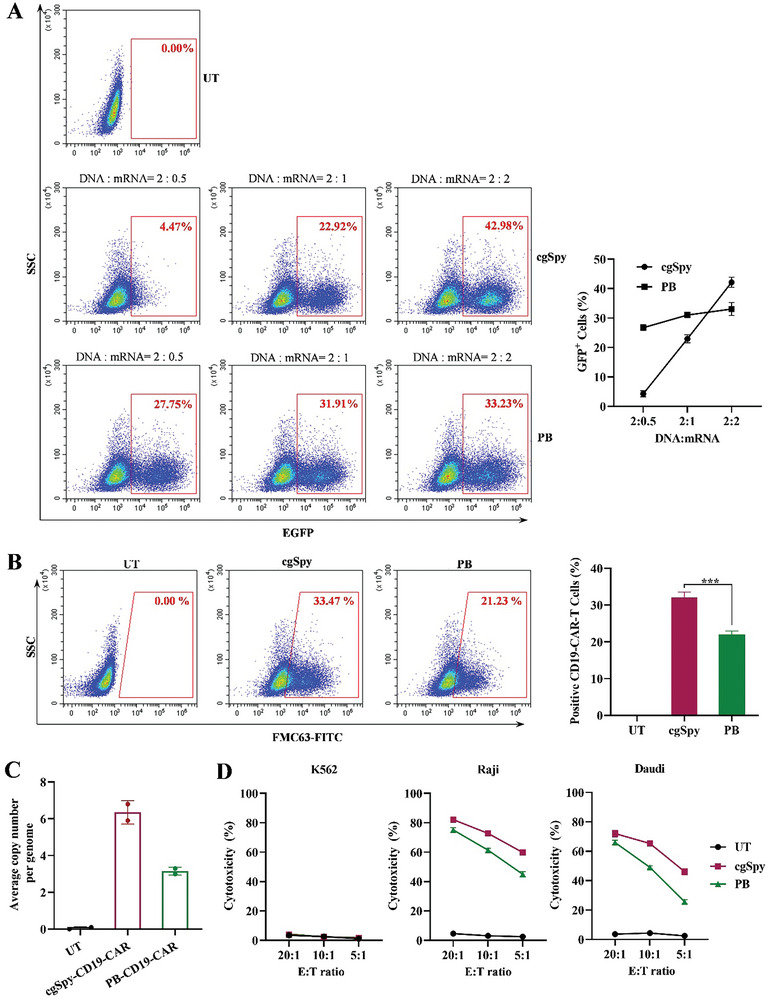
Generation of CD19 CAR T cell by transposon system *cgSpy* and functional analysis of the cell product. A) Transgene efficiency by *cgSpy* transposon system in a human T cell line. One million human Jurkat T cells were electrotransfected with 2 µg plasmid DNA of transposon carrying an EGFP reporter and indicated ratios of transposase mRNA. PiggyBac (*PB*) transposon carrying EGFP served as positive control. Flow cytometry analysis of EGFP+ cells after 11 days of transfection was conducted, and representative flow cytometry plots were shown. B) Production of CD19‐CAR‐T cells by *cgSpy* transposon system. One million activated primary human CD3^+^ T cells isolated from the PBMC of healthy donors were co‐electransfected with 2 µg plasmid DNA of transposon‐loaded CD19‐CAR and 2 µg transposase mRNA, *PB* transposon served as positive control, and untransfected (UT) cells were served as negative control. After 14 days of transfection, flow cytometry analysis of CD19‐CAR positive cells by using FAM63‐FITC antibody was conducted. C) Represents insertion copy number in primary human T cells. Human T cells were treated as in (B). After 10 days of transfection, the average number of CD19‐CAR insertions per human T cell genome was measured using two independent digital droplet PCR assays. D) Cytotoxicity activity of CD19‐CAR T cells generated by *cgSpy* and *PB* transposon system. Cytotoxicity activity was valued by LDH assay after a 5‐h co‐culture of engineering CD19‐CAR T cells with target cells, including human B lymphoma cells, Raji and Daudi. CD19 deficiency K562 lymphoblast served as a negative control. Data were obtained from three independent experiments with different T‐cell donors (*n* = 3 for each group). E:T ratio, effector‐to‐target ratio. Data are shown as mean ± SD. Statistical significance (Studuent's *t*‐test) was measured for the values of the *PB* versus cgSpy as indicated by stars; ^***^ *p* < 0.01.

To further quantify *cgSpy*‐mediated transgenesis in human T cells, we applied EGFP reporter and CD19 chimeric antigen receptor (CAR) systems. First, we want to determine whether the transposon‐mediated transposition depends on the dosage of the transposase. We transfected Jurkat cells with transposon plasmid DNA carrying EGFP reporter and transposase mRNA at a ratio ranging from 2:0.5 to 2:2. The flow cytometry analysis showed that a clear increase of *cgSpy*‐mediated gene transposition with a dose‐dependent of transposase mRNA, while only a slight improvement in *PB* system (**Figure** **7**A). Significantly, at the ratio of 2:2 of transposon DNA related to transposase mRNA, the transposition efficiency of both *cgSpy* and *PB* reaches the highest, and the transposition in the *cgSpy* system is slightly higher than that of *PB* (*hyPB* transposase) (Figure 7A).

To evaluate the applicability of *cgSpy* system‐mediated CAR‐T cell therapy, we co‐transfected primary human T cells with *cgSpy* transposon plasmid that contained a canonical second‐generation CD19 CAR and transposase mRNA at the ratio of 2:2 as described above. The efficiency of cells acquired by the transposon plasmid was evaluated by flow cytometry 2 days after transfection. Both of the transposons, *cgSpy* and *PB*, that carry CD19 CAR were highly expressed, and >90% positive were detected with FMC63‐FITC antibody (an indicator of CD19 CAR, Figure S3, Supporting Information). After 2 weeks of transfection, we observed ≈33% CD19 CAR‐positive cells in *cgSpy* system‐mediated transgenesis, which is relatively higher than the rate with the *PB* system (Figure 7B). Furthermore, we have analyzed the average copy number of CD19‐CAR insertion per genome in *cgSpy* and *PB*‐engineered T cells. The data show more inserts in *cgSpy*‐engineered T cells than *PB* (Figure 7C). Next, we assessed the CAR‐T function by analyzing the cytotoxicity activity of *cgSpy* and *PB*‐engineered T cells against CD19‐positive Raji and Daudi B lymphoma cells and CD19‐negative K562 cells in vitro. CD19 CAR‐T cells showed strong lysis of Raji and Daudi cells, but low killing activity of CD19 negative K562 cells (Figure 7D). Moreover, CD19 CAR‐T cells generated with the *cgSpy* system possess a relatively higher cytotoxicity activity at all ranges of effector‐to‐target (E:T) ratio (Figure 7D). Together, these data indicate that the *cgSpy* transposon can efficiently mediate gene transfer in T cells and is expected to become a new gene delivery tool for CAR‐T therapy.

## 3 Discussion

### 3.1 Evolution of *Spy* and Transposition Activity Comparison

Here, by revisiting the evolution of the *Spy* transposons, which belong to the *PHIS* superfamily, we confirm that this family has a limited distribution in eukaryotes. They invade only three phyla of invertebrates, primarily insects and arachnids. These elements exhibit a simple structural organization, with variable TIRs spanning from 5 to 691 base pairs and one or two open reading frames encoding a transposase of ≈700 amino acids. Upon conducting a bioinformatics analysis, we discovered that the genomes of certain invertebrate species not only display high sequence identities but also have low K divergence, implying a relatively recent invasion by these transposons in these species. Furthermore, some of these genomes may still possess active *Spy* transposons. To evaluate their potential utility, a cell transposition assay was performed. This demonstrated that native *Spy* transposons from the genomes of *C. gigas* and *C. virginica* exhibit promising activity in human cells, compared to *Spy* transposons from *Hedya salicella*, *Cerceris rybyensis*, and *Lymantria dispar* genomes. Moreover, shortening *cgSpy* TIRs did not boost activity as hoped. Engineered variants lagged in HeLa cells, suggesting more to TIR optimization than just length. Deeper exploration is needed to unlock this transposon's full potential. To gain a deeper understanding of the key biological features, namely the OPI, cargo capacity, and insertion preference, the *cgSpy* transposon was further profiled, revealing its unique characteristics.

### 3.2 Absent Overproduction Inhibition Phenomenon of cg*Spy*

Overproduction inhibition is a critical biological feature of cut‐and‐paste DNA transposons. It occurs because the transposon ends become over‐occupied with transposase dimers, which obstruct the assembly of the transpososome.^[^ ^41^ ^]^ This phenomenon has been observed in most well‐defined DNA transposons, including *PS*,^[^ ^32^ ^]^ *SB*,^[^ ^13^ ^]^ *ZB*,^[^ ^31^ ^]^ *Tc1/mariner*,^[^ ^42^ ^]^ *PB*,^[^ ^43^ ^]^ and *Tol2*.^[^ ^36^, ^44^ ^]^ In the present study, we demonstrated that the cg*Spy* transposon does not exhibit the typical OPI behavior in HeLa cells when subjected to both high and low dosages of donor plasmids. This differs from the *PB* and *SB* controls, which both display the OPI phenomenon. Notably, *SB* is more sensitive to OPI than *PB*, as indicated in Figure 3A,B of this study, and *PS*, as previously reported.^[^ ^32^ ^]^ The absence of the OPI phenomenon in the cg*Spy* transposon may be attributed to the presence of long TIRs. These TIRs provide more space for transposase binding during dimerization. Overall, our findings suggest that the *cgSpy* system does not exhibit the typical OPI phenomenon, making it a potent genetic tool for practical applications such as antibody production utilizing cell culture technology. This approach is particularly beneficial when high levels of transgene expression are desired, a goal that can be accomplished by administering an elevated dosage of transposase to augment the number of transgene insertions per cell genome. In contrast, within the realm of human gene therapy, such as chimeric antigen receptor T (CAR‐T) cell therapy, safety is the paramount concern. An excessively high number of transgene copies in the cell genome could increase the risk of disrupting host functional genes.

### 3.3 Insertion Preference Difference

The study investigates *Spy*, a distinct group of eukaryotic DNA transposons that distinguishes itself from other cut‐and‐paste transposons by lacking target TSDs.^[^ ^8^, ^35^ ^]^ Instead, *Spy* transposons integrate precisely between specific AAA and TTT nucleotides in the host genome without altering the host sequences. They achieve this by utilizing blunt‐ended double‐strand breaks at both ends and inserting themselves into non‐staggered positions in the target DNA. This unique integration mechanism sets them apart from other eukaryotic DNA transposons, such as *Tc1/mariner*, *PB*, and certain class 1 transposons, making them valuable for genetic manipulation. The efficiency of transposon systems greatly depends on the integration site, which varies depending on the intended application.^[^ ^22^ ^]^ An ideal transposon system for gene therapy should not strongly prefer inserting into gene bodies. Conversely, transposons that prefer genic regions may be better suited for mutagenesis, while those favoring gene‐regulatory regions may be more suitable for functional genomic analysis, such as enhancer trapping. The *SB* and *ZB* transposons display a nearly random integration profile in the human genome.^[^ ^41^ ^]^ However, *ZB* exhibits a slight preference for gene and gene‐regulatory regions. In contrast, the integration patterns of *PS*, *PB*, *SPIN*, *TcBuster*, and *Tol2* show a notable inclination toward genic regions and transcriptional regulatory regions of genes at the genomic level.^[^ ^31^, ^32^, ^41^, ^45^, ^46^, ^47^ ^]^ Through genome‐wide sequencing of insertion sites, we discovered that the consensus sequences for cg*Spy* integration sites are AAA and TTT. Additionally, our findings reveal that the cg*Spy* transposon system exhibits an integration profile that falls between that of *ZB* and *SB*. It shows a slight preference for integrating into the 5′ ends of regular genes, gene bodies, and cancer genes. Furthermore, it demonstrates a greater inclination toward promoter and/or enhancer regions, as well as histone marks, compared to *ZB* and *SB*. Consistent with these observations, our analyses indicate that the insertion frequencies of cg*Spy* into genomic safe harbors fall between those of *ZB* and *SB*. This suggests that cg*Spy* holds potential for gene therapy purposes.

### 3.4 Cargo Capacity

The ability to transport cargo is essential for transposition, which limits the applicability of the transposon system to transgenesis, particularly for human gene therapy, where large transgenes may be required.^[^ ^30^, ^33^ ^]^ While studies have reported the delivery of very large DNA fragments using *SB*, *PB*, and *Tol2* transposon systems,^[^ ^48^, ^49^ ^]^ the transposition activities of most well‐defined transposons, including *PB*, *SB*, *Tol2*, and *PS*,^[^ ^32^ ^]^ significantly decrease as the insertion size increases. Different transposon systems also have varying tolerances for cargo size, with increasing insertion size impeding transpositional activity. Notably, the *SB* transposon system appears to be more sensitive to cargo size, with a transposition efficiency decrease of ≈30% per kb increase in insertion size.^[^ ^40^ ^]^ Conversely, the *PS* transposon system exhibits a transposition activity decrease of ≈12% per kb increase in insertion size.^[^ ^32^ ^]^

In this study, we observed that *cgSpy* transposons displayed a decrease in transposition efficiency as the cargo size increased, with a decrease of ≈12% per kb increase in insertion size, similar to the *PS* transposon system (38). However, we also noticed that the decrease in transposition activity of *cgSpy* was minimal (only 4%) when the insertion size increased from 1.6 to 3.1 kb, which is significantly different from *SB* ^[^ ^40^ ^]^ and *PS* ^[^ ^32^ ^]^ systems, where they exhibited dramatic drops in transposition activity within the same range. This suggests that the *cgSpy* system may have more potential for human gene therapy when large cargo sizes are required. Furthermore, based on experience with *SB*,^[^ ^50^ ^]^ optimizing the TIR structure could enhance *cgSpy*’s cargo capacity. Additionally, it is valuable to engineer *cgSpy* transposase as a genetic tool with relatively high activity for diverse purposes.

### 3.5 Application Potential of *cgSpy* in the CAR‐T Therapy

Transposon‐based CAR‐T cell therapy is a novel and promising approach to cancer treatment.^[^ ^51^ ^]^ Compared to traditional lentivirus‐based CAR‐T cells, transposon‐based CAR‐T cells have several advantages. Transposon vectors are less immunogenic and less likely to cause insertional mutagenesis than lentiviral vectors.^[^ ^51^ ^]^ More importantly, the transposon system can overcome the high production cost and transgene capacity limitations, offering the possibility for wider use and application. Transposon‐based CAR‐T cell therapy holds great promise for cancer treatment and could eventually become a standard of care for certain types of cancer.^[^ ^52^ ^]^ As for earlier transposon systems like *PB* and *SB*, many reports describe their application in CAR‐T therapy,^[^ ^53^, ^54^ ^]^ demonstrating excellent results comparable to viral‐based methods. However, the OPI effect limits further improvements in the transgene efficiency of *PB* and *SB* transposons. The *cgSpy* transposon we discovered shows no apparent inhibitory effect, indicating its great potential as a gene delivery tool. The transposition efficiency of *cgSpy* has a clear concentration dependence on the use of transposase. Nevertheless, *cgSpy* exhibited significantly lower transposition efficiency when fewer amounts of transposase mRNA were used, possibly due to its relatively low enzymatic activity. In the future, we will screen *cgSpy* transposase variants with higher activity through site‐directed mutagenesis to improve its integration efficiency into the genome.

Recently developed new biotechnologies, such as mRNA synthesis and minicircle vector design, have the potential to further increase gene delivery stability and efficiency. Our future goal is to combine these technologies to develop the next generation of *cgSpy* transposon system‐based CAR‐T engineering. It is noteworthy that a clinical trial investigating product‐derived lymphoma following infusion of piggyBac‐modified CD19 CAR‐T cells yielded negative results.^[^ ^55^ ^]^ One patient developed CAR T‐cell‐derived lymphoma, and unfortunately, another patient died. This case underscores the need for caution when using novel gene transfer methods to create genetically modified immune cell therapies. Further animal research and clinical trials are necessary to fully evaluate the potential benefits of *cgSpy* transposon‐based applications in CAR‐T therapy. Furthermore, engineers have designed a fusion between transposase and CRISPR‐associated (Cas) proteins to improve the specificity of transposon‐mediated gene targeting. Both *SB* and *PB* transposases have been successfully combined with Cas9, resulting in a retargeting capability for transposon integration.^[^ ^56^, ^57^ ^]^ Investigating the fusion of *Spy* transposase with RNA‐guided nucleases, including Cas9, Cas12, or miniature nucleases (such as IscB and TnpB), for safer genomic integration, represents a valuable direction for future research.

## 4 Conclusion

To summarize, this study demonstrates that *cgSpy* is a relatively recent invader of the *C. gigas* genome. In mammalian cells, it exhibits significant transposition activity. Its integration profile and insertion frequencies into genomic safe harbors fall between those of *ZB* and *SB* transposons. Additionally, the *cgSpy* transposon system shows enhanced tolerance for cargo size. Furthermore, the *cgSpy* transposon system can effectively facilitate gene transfer in T cells and mediate CAR‐T cytotoxicity activity. These findings indicate that the *cgSpy* transposon serves as a powerful and efficient alternative gene transfer tool for human gene therapy and transgenesis.

## 5 Experimental Section

### *Spy* Transposon Mining

To determine the presence and distribution of *Spy* transposons in various genomes, *Spy* transposases sourced from related species as a reference were utilized for a search in the whole‐genome shotgun contig database (WGS) at NCBI using TblastN with an E‐value threshold of 1^e‐100^. Subsequently, the presence of *Spy* transposons in a particular species was manually confirmed upon identification of the catalytic domain (DDE). Following this confirmation, related transposases were used as queries to identify more specific transposons. For further analysis, significant hits were extracted with 2000 bp flanking sequences and aligned using ClustalW within BioEdit to determine their boundaries.^[^ ^13^, ^58^ ^]^ To estimate copy numbers, all hits obtained from the WGS genome database were used that had >40% coverage in length and >80% identity. For species with fewer than five full‐length copies, the representative sequence containing the complete TIRs was selected and encoded an intact transposase protein (>500 amino acids containing an intact DNA‐binding domain (DBD) and DDE domains). The PSIPRED program was used to predict the secondary structure of the transposase (http://bioinf.cs.ucl.ac.uk/psipred/),^[^ ^59^ ^]^ while the online hmmscan web server was used to identify protein domains using hidden Markov models (https://www.ebi.ac.uk/Tools/hmmer/search/hmmscan).^[^ ^60^ ^]^

### Evolutionary Relationships of *PHIS* Superfamily

To determine the evolutionary lineage of these families, the conserved DDE domains of *Spy* transposases were aligned with representative families from the *PHIS* superfamily and IS5 transposases from prokaryotes (used as an outgroup) using MAFFT v.7.310.^[^ ^8^, ^35^, ^61^ ^]^ From this comparison, a phylogenetic tree was inferred based on the ≈300 amino acids of the conserved DDE domain using the maximum likelihood method with IQ‐TREE.^[^ ^62^ ^]^ The best model was selected by ModelFinder, embedded in the IQ‐TREE program, and the reliability of the maximum likelihood tree was determined using a 1000‐replicate ultrafast bootstrap approach.^[^ ^63^ ^]^ To deduce the evolutionary dynamics of *Spy* within genomes, the calcDivergenceFromAlign.pl package was employed from RepeatMasker,^[^ ^64^ ^]^ which computed the Kimura two‐parameter distance (K divergence).^[^ ^65^ ^]^

### Confirmation of Transposition Activity and Design of Plasmids

Codon optimization of transposase genes from *Colletes gigas*, *Crassostrea virginica*, *Hedya salicella*, *Cerceris rybyensis*, and *Lymantria dispar* was undertaken to improve their efficacy in human cells. The Integrated DNA Technologies (IDT) database (https://login.idtdna.com/CodonOpt) was employed for this optimization. Subsequently, GenScript (USA) synthesized the codon‐optimized transposase sequences, which were then cloned into transposase expression plasmids as detailed elsewhere.^[^ ^36^ ^]^ These resulting recombinant vectors (also named helper plasmids) were pCAG‐*cgSpy*, pCAG‐*cvSpy*, pCAG‐*hsSpy*, pCAG‐*crSpy*, and pCAG‐*ldSpy*. Furthermore, TIR sequences from the genomes of *Crassostrea virginica*, *Hedya salicella*, and *Lymantria dispar* were obtained through the annealing of oligos, and TIR sequences from the genomes of *Colletes gigas* and *Cerceris rybyensis* were synthesized (GenScript, USA), followed by cloning into the pLB vector (TIANGEN, Beijing, China) and sequencing. The resultant vectors were labeled as pcg*Spy*TIR, pcv*Spy*TIR, phs*Spy*TIR, pcr*Spy*TIR, and pld*Spy*TIR. Then, the donor plasmids, namely pcg*Spy*‐PGK‐Neo, pcv*Spy*‐PGK‐Neo, phs*Spy*‐PGK‐Neo, pcr*Spy*‐PGK‐Neo and pld*Spy*‐PGK‐Neo, were created by incorporating the PGK‐Neo cassette into pcg*Spy*TIR, pcv*Spy*TIR, phs*Spy*TIR, pcr*Spy*TIR and pld*Spy*TIR, respectively, via the NruI site. The UTRs (the sequences between the TIR and the ORF) were synthesized (GenScript, USA), together with short TIRs and included in the donor vectors (named as *cvSpy*TIR‐UTR‐PGK‐Neo, *hsSpy*TIR‐UTR‐PGK‐Neo, *crSpy*TIR‐UTR‐PGK‐Neo, and *ldSpy*TIR‐UTR‐PGK‐Neo). For evaluating the *cgSpy*TIR in T cell engineering, an EGFP reporter fragment or a second‐generation CD19‐CAR expression fragment was subcloned into *cgSpy*TIR, named *cgSpy*‐EGFP and *cgSpy*‐CD19‐CAR, respectively. A list of all the primers employed in this process can be found in Table S3 (Supporting Information). The highly active transposases of *SB* (*SB100X*) and *PB* (*hyPB*) transposons, which were engineered to improve the transposition activity based on the original active transposases,^[^ ^37^, ^39^ ^]^ were applied in cell tests as controls. All vector sequences are available upon reasonable request.

### Optimizing *cgSpy* Transposon TIRs Through Vector Design

The versatility of the *cgSpy* transposon was investigated by engineering four variants aimed at optimizing its TIRs. The TIRs of *cgSpy* were engineered using an alignment of *Spy* transposon TIRs derived from various species. Starting with the wild‐type *cgSpy* (wtL+wtR), with both TIRs spanning 467 bp, sequence analysis of related *Spy* transposons guided the design of shorter, potentially more efficient TIRs. Four modifications resulted: L1+wtR retained the wtR sequence but replaced wtL with a 320 bp fragment (L1); L2+wtR similarly swapped wtL for a shorter 191 bp fragment (L2); wtL+R1 kept wtF but exchanged wtR for a 320 bp sequence (R1); and wtL+R2 maintained wtL while incorporating the even shorter 191 bp R2. Standard molecular cloning techniques involving PCR amplification with flanking primers containing restriction enzyme sites, digestion, and ligation into a vector were employed to construct these variants (Figure 3A). Sanger sequencing confirmed the presence of the desired modified TIRs in the resulting plasmids. This approach not only provides insights into TIR optimization but also lays the groundwork for further functional characterization of these *cgSpy* variants. The primers used for the *cgSpy* TIRs optimization are listed in Table S3 (Supporting Information).

### Vectors Used to Assess the Cargo Capacity of the *cgSpy* Transposon

To gauge the cargo capacity, a series of donor plasmids containing fragments of different sizes were created. Initially, the p*cgSpy*‐PGK‐Neo donor plasmid was used, which already contained a 1.6 kb fragment. EcoRI was used to cut this plasmid, and λ‐phage genome fragments of 1.5, 3.5, and 6.9 kb were ligated between poly(A) and the TIR sequences to generate p*cgSpy*‐PGK‐Neo/3.1/5.1/8.5. To meet the donor plasmid size requirement, λ‐phage DNA fragments of 5.4, 3.4, and 6.9 kb were used as stuffer material and cloned into p*cgSpy*‐PGK‐Neo/1.6/3.1/5.1 via the HindIII site outside of the transposon TIRs. All stuffers were PCR‐amplified from λ genomic DNA, and their respective primers are listed in Table S3 (Supporting Information).

### Cell Culture, Transfection, and Electrotransfection

The HeLa, HepG2, Jurkat, K562, Raji, and Daudi cell lines, were initially sourced from ATCC in the USA. HeLa and HepG2 were cultured in Dulbecco's modified Eagle's medium (DMEM; Gibco, USA) supplemented with 10% fetal bovine serum (FBS; Gibco, USA) and 1% penicillin‐streptomycin (PS; Gibco, USA). Jurkat, K562, Raji, and Daudi cell lines were cultured in RIPM‐1640 (Gibco, USA) supplemented with 10% FBS and 1% PS. Furthermore, they were kept in a CO_2_ incubator at 37 °C. The instructions of the FuGENE HD Transfection Reagent (Promega, USA) were followed to execute the transfection process. On the day before the transfection, 3 × 10^5^ cells were placed onto each well of six‐well plates to ensure that the cells had an ≈80% confluency on the day of transfection. Co‐transfection with donor plasmids and transposase‐expressing helper plasmids was done using a reagent‐to‐DNA ratio of 3:1. The transfection assay underwent three repetitions per group.

Human peripheral blood mononuclear cells (hPBMC) of healthy donors with informed consent were obtained from Milestone Biotechnologies (Shanghai, China). Primary human T cells were isolated from hPBMC with a negative selection method according to the instructions of EasySep Human T Cell Isolation Kit (StemCell, Canada). T cells were cultured in ImmunoCult‐XF T cell expansion medium (StemCell, Canada) with 50 ng mL^−1^ recombinant human IL‐2 (R&D Systems, USA). T cells were activated with ImmunoCult Hu CD3/CD28 T Cell Act (StemCell, Canada) for 2 days. On day 2, the CD3/CD28 T Cell Act was removed, and the activated T cells were washed with sterile PBS twice and prepared for electrotransfection by the Neon transfection system (ThermoFisher Scientific, USA). For electrotransfection, 1 × 10^6^ activated T cells were suspended in 100 µL T buffer or 1 × 10^6^ Jurkat cells suspended in 100 µL R buffer from the Neon transfection Kit (ThermoFisher Scientific, USA), then pre‐mixed with the indicated amount of DNA/mRNA and transferred to electroporation cuvettes with no bubble. The electrotransfection parameters are set as follows, primary T cells (2300 V, 3 ms, and 4 pulses) and Jurkat cells (1700 V, 20 ms, and 1 pulses), respectively. Untreated (UT) cells were used as a negative control. After electrotransfection, the cells were returned to the expansion medium and maintained at a concentration of 1 × 10^6^ T cells mL^−1^.

### Flow Cytometry

To evaluate the efficiency of transgene mediated by the *cgSpy* or *PB* transposon system, the transposon plasmids carrying an EGFP expression cassette and the indicated concentration of transposase mRNA were pre‐mixed and electrotransfected into Jurkat cells. After 14 days of expansion, the integration efficiency of transgene was verified by detecting the EGFP expression using the Beckman Coulter Cytoflex S flow cytometer (Beckman Coulter, USA). To verify the CD19‐CAR expressed on the primary human T cells, activated CD3^+^ T cells were electrotransfected with the plasmid DNA of transposon‐TIR‐flamed CD19‐CAR and transposase mRNA at a ratio of 2:2, like that 2 ug DNA and 2 ug mRNA for 1 × 10^6^ cells. At 11 days after electrotransfection, 2 × 10^5^ human T cells were collected and washed with PBS twice, and incubated with FMC63‐scFv‐FITC antibody (ACROBiosystems, USA) on the ice for 30 min. After that, the stained T‐cell samples were washed once to remove the unbounded antibody and measured by using the flow cytometer. The data were analyzed with the CytExpert 2.3 software (Beckman Coulter, USA).

### Cytotoxic Activity Analysis

To measure the CD19‐CAR‐T cells cytotoxicity, target cell lines, including CD19^‐^ K562, CD19^+^ Raji, and CD19^+^ Daudi cells, were incubated with the effector T cells, *cgSpy*‐CD19‐CAR‐T cells, or *PB*‐CD19‐CAR‐T cells or UT T cells, at an effector‐to‐target (E:T) ratio ranging from 20:1 to 5:1. The target cells cultured without CAR‐T cells treated with lactate dehydrogenase (LDH) releasing regent (Beyotime Biotechnology, China) served as the sets of maximum enzyme activity of target cells. The target cells cultured only without any treatment served as blank control. After 5 h of inoculation, the cytotoxic activity was assessed using the LDH assay following the manufacturer's instructions (Beyotime Biotechnology, China). Cytotoxicity was calculated as (absorbance of the treated sample – absorbance of control sample) / (absorbance of maximum enzyme activity of cells – absorbance of control sample) × 100.

### Transpositional Activity Assay, Overproduction Inhibition, and Cargo Capacity Evaluation

To evaluate the transpositional activity of *cgSpy*, the following procedure was used. HeLa cells were co‐transfected with 500 ng of donor plasmids and 500 ng of helper plasmids. After 48 h, 0.5% of the cells were replated in 6‐cm plates and cultured in a medium containing 600 µg mL^−1^ G418 (Gibco, USA) for selection. After two weeks of drug selection, the resistant colonies were stained with Giemsa reagent (Promega, USA) and counted using ImageJ. The previously established methodology^[^ ^34^ ^]^ was utilized to conduct the overproduction inhibition assay. Varying amounts of helper plasmids (ranging from 0 to 1500 ng) were co‐transfected with 10 or 500 ng of donor plasmids. The transfection reaction included the addition of the empty vector pCAG to fulfill a total amount. Cells were trypsinized and re‐plated on 6‐cm plates after 48 h of transfection. In cases where 10 ng of donor plasmids were used, 5% of the transfected cells were seeded, whereas only 0.5% were seeded for 500 ng of donor plasmids. The cultures were then grown under selection in a 600 ug mL^−1^ G418 medium for two weeks. G418‐resistant colonies were visualized by Giemsa staining, and statistical analysis of the colony numbers was performed using ImageJ. Each sample was prepared as a triple replicate in three individual experiments. To determine the cargo capacity of *cgSpy*, 1130 ng of the donor plasmids carrying fragments of different sizes and 1000 ng of helper plasmid were co‐transfected into HeLa cells. After two weeks of G418 selection, the colonies were counted by ImageJ. In the cargo capacity assay, each group underwent three repetitions.

### Staining and Enumeration of Cells

Following a 14‐day selection process utilizing G418, the colonies were manually stained using the Giemsa reagent. The process consisted of washing the cell colonies with phosphate‐buffered saline (PBS) and subsequent treatment using a 1:1 ratio of PBS and methanol at room temperature for 2 min. The cells were then fixed with methanol for 10 min, and then stained with the Giemsa reagent for 2 min. Finally, the cells were immersed in a tenfold diluted Giemsa reagent for ten more minutes before scanning and counting the stained cells using the ImageJ software.

### Integration Site Analysis

The transposon insertion libraries were constructed according to the method.^[^ ^31^, ^32^ ^]^ In brief, the HepG2 cells were co‐transfected with donor and helper plasmids and selected with 900ug/ml G418 in 10‐cm dishes for two weeks. Then ≈30 000 G418‐resistent colonies were collected and the genomic DNA was extracted. 1.5 ug DNA was sheared using a Covaris M220 instrument to achieve a median length of 500 ± 50 bp. The resulting DNA was purified using AmpureXP beads, and a target gene‐specific biotinylated primer was used for primer extension. The extension product was then filtered and captured using Dynabeads M280 (Invitrogen, USA), followed by ligation to linker cassettes with a molecular barcode. The ligation product underwent the first round of PCR with biotinylated target gene‐ and linker‐specific primers. Biotinylated PCR products were captured and pooled, as a template for the second round PCR using specific primers with barcodes. The second PCR product as an insertion library underwent deep sequencing by Miseq EQ (Illumina, USA). Table S3 (Supporting Information) provides the primer sequences used.

The sequencing data was analyzed using the Genome Integration Site Analysis Pipeline (GENE‐IS),^[^ ^66^ ^]^ and specifications for the raw read processing and mapping parameters were already established.^[^ ^66^ ^]^ To produce high‐quality insertion sites, the raw data went through these procedures and criteria: First, the raw paired‐end sequencing data were filtered by examining if the initial 100 bp of Forward reads had a megaprimer sequence and if the first 100 bp of Reverse read contained a linker cassette sequence in every paired‐end series. Next, reads lacking sample‐specific barcode sequences were filtered out, and Skewer (version 0.1.117)^[^ ^67^ ^]^ trimmed primers, linkers, and anchor sequences from the remaining reads. Reads below 80 bp after trimming were eliminated. The filtered reads were mapped to the human genome (hg38) with BWA software to recognize the insertion sites. For reads with multiple alignment loci on the human genome, the best mapping record was obtained as an insertion site via a secondary alignment using BLAT software (version 3). Lastly, insertion sites with support from no less than 10 independent reads at the target site of the human genome (hg38) were chosen as the final output.

The genic features and epigenomic annotation of the integration sites were referred to in the previous study.^[^ ^20^ ^]^ The coordinates for genic features were obtained from the UCSC Table Browser (https://genome.ucsc.edu/cgi‐bin/hg), and the list of cancer genes was downloaded from the homepage (http://www.bushmanlab.or/lingks/genelists). The ChIP‐Seq peaks of various histone modifications in HepG2 cells were downloaded from the ENCODE project homepage (https://www.encodeproject.org/) via the wgEncodeBroadHistoneHepg2 dataset. The Genomation package was applied to examine the representation of the insertion sites in various genic categories.^[^ ^20^ ^]^ The insertion site frequencies for cg*Spy*, *ZB*, and within these regions were compared with 100000 randomly generated loci in the human genome. File S1 provides coordinates for *cgSpy* insertions within the human genome.

### Insertion Copy Number of *cgSpy*

Genomic DNA from the Neo gene group and CD19‐CAR gene group were extracted using the TIANamp Genomic DNA kit (TIANGEN, China). The average number of neomycin‐resistance transgene or CD19‐CAR gene insertions was measured by digital droplet PCR as follows: 200 ng genomic DNA was digested with 20 units of DpnI restriction enzyme (NEB, USA) in 30 uL final reaction volume overnight to eliminate non‐integrated transposon plasmid DNA. Then, the DNA from the Neo gene group was fragmented with CviQI (NEB, USA) for 4 h at 25 °C and it from the CD19‐CAR gene group was digested by BfaI enzyme for 4 h at 37 °C. The samples were subjected to PCR amplification using probes and primers for the Neo (or CD19‐CAR) gene and for the single‐copy human RPP30 gene to measure the genome count.^[^ ^68^ ^]^ The PCR reactions were performed in 20 final volumes with 10 ng of fragmented gDNA using the ddPCR Supermix for Probes (No dUTP) master mix (Bio‐rad, USA) with 900 nm primers and 250 nm probes (for the sequence of primers and probes see Table S3, Supporting Information). The PCR droplets were generated in the QX200 device (Bio‐rad, USA). The PCR program was 95 °C 10 min; 40 cycles of 94 °C 10s, 60 °C 1 min; 98 °C 10 min. After thermal cycling, the fluorescent droplets were counted in the QX200 Droplet Reader (Bio‐rad), and genomic copy numbers were calculated with the Quanta Soft Software (Bio‐rad, USA).

## Conflict of Interest

The authors declare no conflict of interest.

## Supporting information

Supporting Information

## Data Availability

The data that support the findings of this study are available in the supplementary material of this article.

## References

[1] C. Gilbert , J. Peccoud , R. Cordaux , Annu. Rev. Entomol. 2021, 66, 355. 32931312 10.1146/annurev-ento-070720-074650

[2] R. N. Platt , M. W. Vandewege , D. A. Ray , Chromosome Res. 2018, 26, 25. 29392473 10.1007/s10577-017-9570-z PMC5857283

[3] K. K. Kojima , Genes Genet. Syst. 2019, 94, 233. 10.1266/ggs.18-00024 30416149

[4] B. Piégu , S. Bire , P. Arensburger , Y. Bigot , Mol. Phylogenet. Evol. 2015, 86, 90. 25797922 10.1016/j.ympev.2015.03.009

[5] R. Craigie , K. Mizuuchi , Cell 1985, 41, 867. 2988793 10.1016/s0092-8674(85)80067-2

[6] W. FitzHugh , R. Funke , D. Gage , K. Harris , A. Heaford , J. Howland , L. Kann , J. Lehoczky , R. LeVine , P. McEwan , K. McKernan , J. Meldrim , J. P. Mesirov , C. Miranda , W. Morris , J. Naylor , C. Raymond , M. Rosetti , R. Santos , A. Sheridan , C. Sougnez , E. S. Lander , L. M. Linton , B. Birren , C. Nusbaum , M. C. Zody , J. Baldwin , K. Devon , K. Dewar , M. Doyle , et al., Nature 2001, 15, 860.

[7] X. Zhang , C. Feschotte , Q. Zhang , N. Jiang , W. B. Eggleston , S. R. Wessler , Proc. Natl. Acad. Sci. 2001, 98, 12572. 11675493 10.1073/pnas.211442198 PMC60095

[8] M.‐J. Han , H.‐E. Xu , H.‐H. Zhang , C. Feschotte , Z. Zhang , Genome. Biol. Evol. 2014, 6, 1748. 24966181 10.1093/gbe/evu140 PMC4122938

[9] D. Lisch , Trends Plant Sci. 2002, 7, 498. 12417150 10.1016/s1360-1385(02)02347-6

[10] C. P. Marquez , E. J. Pritham , Genetics 2010, 185, 1507. 20457878 10.1534/genetics.110.116673 PMC2927773

[11] J. Jurka , V. V. Kapitonov , A. Pavlicek , P. Klonowski , O. Kohany , J. Walichiewicz , Cytogenet. Genome Res. 2005, 110, 462. 16093699 10.1159/000084979

[12] Y.‐W. Yuan , S. R. Wessler , Proc. Natl. Acad. Sci. 2011, 108, 7884.21518873

[13] Z. Izsvák , Z. Ivics , Molecular Therapy 2004, 9, 147. 14759798 10.1016/j.ymthe.2003.11.009

[14] D. R. Kim , Y. Dai , C. L. Mundy , W. Yang , M. A. Oettinger , J. Recombinase Genes Dev. 1999, 13, 3070. 10601033 10.1101/gad.13.23.3070 PMC317176

[15] M. A. Landree , J. A. Wibbenmeyer , D. B. Roth , J. Recombination Genes Dev. 1999, 13, 3059. 10601032 10.1101/gad.13.23.3059 PMC317185

[16] M. S. Junop , D. B. Haniford , EMBO J. 1997, 16, 2646. 9184211 10.1093/emboj/16.10.2646 PMC1169875

[17] M. Katzman , M. Sudol , J. Virol. 1995, 69, 5687. 7637015 10.1128/jvi.69.9.5687-5696.1995 PMC189427

[18] R. S. Appa , C.‐G. Shin , P. Lee , S. A. Chow , J. Biol. Chem. 2001, 276, 45848. 11585830 10.1074/jbc.M107365200

[19] A. L. Harper , M. Sudol , M. Katzman , DNA J. Virol. 2003, 77, 3838. 12610159 10.1128/JVI.77.6.3838-3845.2003 PMC149511

[20] C. Miskey , L. Kesselring , I. Querques , G. Abrusán , O. Barabas , Z. Ivics , Nucleic Acids Res. 2022, 50, 2807. 35188569 10.1093/nar/gkac092 PMC8934666

[21] A. Palazzo , R. M. Marsano , Crit. Rev. Biotechnol. 2021, 41, 792. 33622117 10.1080/07388551.2021.1888067

[22] K. Kawakami , D. A. Largaespada , Z. Ivics , Trends Genet. 2017, 33, 784. 28888423 10.1016/j.tig.2017.07.006 PMC5682939

[23] G. Bourque , K. H. Burns , M. Gehring , V. Gorbunova , A. Seluanov , M. Hammell , M. Imbeault , Z. Izsvák , H. L. Levin , T. S. Macfarlan , D. L. Mager , C. Feschotte , Genome Biol. 2018, 19, 199. 30454069 10.1186/s13059-018-1577-z PMC6240941

[24] J. Sakai , N. Kleckner , Cell 1997, 89, 205. 9108476 10.1016/s0092-8674(00)80200-7

[25] S. Prommersberger , M. Reiser , J. Beckmann , S. Danhof , M. Amberger , P. Quade‐Lyssy , H. Einsele , M. Hudecek , H. Bonig , Z. Ivics , Gene Ther. 2021, 28, 560. 33846552 10.1038/s41434-021-00254-w PMC8455317

[26] Y. Zhang , Z. Zhang , Y. Ding , Y. Fang , P. Wang , W. Chu , Z. Jin , X. Yang , J. Wang , J. Lou , Q. Qian , J. Cancer Res. Clin. Oncol. 2021, 147, 3725. 34032893 10.1007/s00432-021-03613-7 PMC11801842

[27] S. A. Srour , H. Singh , J. McCarty , E. De Groot , H. Huls , G. Rondon , M. Qazilbash , S. Ciurea , G. Bardelli , J. Buck , A. Alousi , Y. Nieto , K. Rezvani , D. Marin , U. Popat , C. Hosing , E. J. Shpall , W. G. Wierda , H. Kantarjian , R. E. Champlin , L. J. Cooper , P. Kebriaei , Blood 2020, 135, 862. 31961918 10.1182/blood.2019002920 PMC8212349

[28] L.‐T. Nguyen , H. A. Schmidt , A. von Haeseler , B. Q. Minh , Phylogenies Mol. Biol. Evol. 2015, 32, 268. 25371430 10.1093/molbev/msu300 PMC4271533

[29] M. Bouallègue , J. D. Rouault , A. Hua‐Van , M. Makni , P. Capy , Genome Biol. Evol. 2017, 9, 323. 10.1093/gbe/evw292 PMC5381638 28082605

[30] P. Kebriaei , Z. Izsvák , S. A. Narayanavari , H. Singh , Z. Ivics , Trends Genet. 2017, 33, 852. 28964527 10.1016/j.tig.2017.08.008

[31] D. Shen , C. Song , C. Miskey , S. Chan , Z. Guan , Y. Sang , Y. Wang , C. Chen , X. Wang , F. Müller , et al., Nucleic Acids Res. 2021, 49, 2126. 33638993 10.1093/nar/gkab045 PMC7913693

[32] S. Wang , B. Gao , C. Miskey , Z. Guan , Y. Sang , C. Chen , X. Wang , Z. Ivics , C. Song , Nucleic Acids Res. 2023, 51, 1843. 36688327 10.1093/nar/gkad005 PMC9976928

[33] Z. Ivics , M. A. Li , L. Mátés , J. D. Boeke , A. Nagy , A. Bradley , Z. Izsvák , Nat. Methods 2009, 6, 415. 19478801 10.1038/nmeth.1332 PMC2867038

[34] I. Grabundzija , M. Irgang , L. Mátés , E. Belay , J. Matrai , A. Gogol‐Döring , K. Kawakami , W. Chen , P. Ruiz , M. K. L. Chuah , et al., Molecular Therapy 2010, 8, 1200. 10.1038/mt.2010.47 PMC2889740 20372108

[35] M.‐J. Han , C.‐L. Xiong , H.‐B. Zhang , M.‐Q. Zhang , H.‐H. Zhang , Z. Zhang , Mob DNA 2015, 6, 12. 26120370 10.1186/s13100-015-0043-7 PMC4482050

[36] I. Grabundzija , M. Irgang , L. Mátés , E. Belay , J. Matrai , A. Gogol‐Döring , K. Kawakami , W. Chen , P. Ruiz , M. K. L. Chuah , T. VandenDriessche , Z. Izsvák , Z. Ivics , Molecular Therapy 2010, 18, 1200. 20372108 10.1038/mt.2010.47 PMC2889740

[37] K. Yusa , L. Zhou , M. A. Li , A. Bradley , N. L. Craig , Proc. Natl. Acad. Sci. 2011, 108, 1531. 21205896 10.1073/pnas.1008322108 PMC3029773

[38] G. Liu , E. L. Aronovich , Z. Cui , C. B. Whitley , P. B. Hackett , J. Gene Med. 2004, 6, 574. 15133768 10.1002/jgm.486 PMC1865527

[39] L. Mátés , M. K. L. Chuah , E. Belay , B. Jerchow , N. Manoj , A. Acosta‐Sanchez , D. P. Grzela , A. Schmitt , K. Becker , J. Matrai , L. Ma , E. Samara‐Kuko , C. Gysemans , D. Pryputniewicz , C. Miskey , B. Fletcher , T. VandenDriessche , Z. Ivics , Z. Izsvák , Nat. Genet. 2009, 41, 753. 19412179 10.1038/ng.343

[40] Z. Izsvák , Z. Ivics , R. H. Plasterk , J. Mol. Biol. 2000, 302, 93. 10964563 10.1006/jmbi.2000.4047

[41] A. Gogol‐Döring , I. Ammar , S. Gupta , M. Bunse , C. Miskey , W. Chen , W. Uckert , T. F. Schulz , Z. Izsvák , Z. Ivics , Cells Molecular Therapy 2016, 24, 592. 26755332 10.1038/mt.2016.11 PMC4786924

[42] A. R. Lohe , D. L. Hartl , Mol. Biol. Evol. 1996, 13, 549. 8882498 10.1093/oxfordjournals.molbev.a025615

[43] S. C.‐Y. Wu , Y.‐J. J. Meir , C. J. Coates , A. M. Handler , P. Pelczar , S. Moisyadi , J. M. Kaminski , Proc. Natl. Acad. Sci. 2006, 103, 15008. 17005721 10.1073/pnas.0606979103 PMC1622771

[44] J. Tipanee , Y. C. Chai , T. VandenDriessche , M. K. Chuah , Biosci. Rep. 2017, 37, BSR20160614. 29089466 10.1042/BSR20160614 PMC5715130

[45] L. E. Woodard , X. Li , N. Malani , A. Kaja , R. H. Hice , P. W. Atkinson , F. D. Bushman , N. L. Craig , M. H. Wilson , PLoS One 2012, 7, e42666. 23166581 10.1371/journal.pone.0042666 PMC3499496

[46] X. Li , H. Ewis , R. H. Hice , N. Malani , N. Parker , L. Zhou , C. Feschotte , F. D. Bushman , P. W. Atkinson , N. L. Craig , Proc. Natl. Acad. Sci. 2013, 110, E478. 23091042 10.1073/pnas.1121543109 PMC3568352

[47] X. Huang , H. Guo , S. Tammana , Y.‐C. Jung , E. Mellgren , P. Bassi , Q. Cao , Z. J. Tu , Y. C. Kim , S. C. Ekker , X. Wu , S. M. Wang , X. Zhou , Molecular Therapy 2010, 18, 1803. 20606646 10.1038/mt.2010.141 PMC2951558

[48] M. L. Suster , K. Sumiyama , K. Kawakami , BMC Genomics 2009, 10, 477. 19832998 10.1186/1471-2164-10-477 PMC2768751

[49] M. Rostovskaya , J. Fu , M. Obst , I. Baer , S. Weidlich , H. Wang , A. J. H. Smith , K. Anastassiadis , A. F. Stewart , Nucleic Acids Res. 2012, 40, e150. 22753106 10.1093/nar/gks643 PMC3479164

[50] H. Zayed , Z. Izsvák , O. Walisko , Z. Ivics , Molecular Therapy 2004, 9, 292. 14759813 10.1016/j.ymthe.2003.11.024

[51] A. Kansagra , S. Farnia , N. Majhail , Am. Soc. Clin. Oncol. Educ. Book 2020, 40, 1. 10.1200/EDBK_279151 32347759

[52] S.‐K. Tey , G. Dotti , C. M. Rooney , H. E. Heslop , M. K. Brenner , Cell Transplantation Biology of Blood, Marrow Transplantation 2007, 13, 913. 17640595 10.1016/j.bbmt.2007.04.005 PMC2040267

[53] M. Suematsu , S. Yagyu , N. Nagao , S. Kubota , Y. Shimizu , M. Tanaka , Y. Nakazawa , T. Imamura , Front Immunol. 2022, 13. 10.3389/fimmu.2022.770132 PMC8829551 35154098

[54] M. Holstein , C. Mesa‐Nuñez , C. Miskey , E. Almarza , V. Poletti , M. Schmeer , E. Grueso , J. C. Ordóñez Flores , D. Kobelt , W. Walther , M. K. Aneja , J. Geiger , H. B. Bonig , Z. Izsvák , M. Schleef , C. Rudolph , F. Mavilio , J. A. Bueren , G. Guenechea , Z. Ivics , Molecular Therapy 2018, 26, 1137. 29503198 10.1016/j.ymthe.2018.01.012 PMC6079369

[55] N. Dong , F. Castillo Tokumori , L. Isenalumhe , Y. Zhang , A. Tandon , T. C. Knepper , H. Shao , L. Zhang , L. Sokol , Blood 2021, 138, 1391. 33819354 10.1002/ajh.26183

[56] A. Kovač , C. Miskey , M. Menzel , E. Grueso , A. Gogol‐Döring , Z. Ivics , Elife 2020, 9, e53868. 32142408 10.7554/eLife.53868 PMC7077980

[57] B. E. Hew , R. Sato , D. Mauro , I. Stoytchev , J. B. Owens , Synth. Biol. 2019, 4. 10.1093/synbio/ysz018 PMC6642342 31355344

[58] R. Kalendar , B. Khassenov , Y. Ramankulov , O. Samuilova , K. I. Ivanov , Genomics 2017, 109, 312. 28502701 10.1016/j.ygeno.2017.05.005

[59] L. J. McGuffin , K. Bryson , D. T. Jones , Bioinformatics 2000, 16, 404. 10869041 10.1093/bioinformatics/16.4.404

[60] S. C. Potter , A. Luciani , S. R. Eddy , Y. Park , R. Lopez , R. D. Finn , Nucleic Acids Res. 2018, 46, W200. 29905871 10.1093/nar/gky448 PMC6030962

[61] K. D. Yamada , K. Tomii , K. Katoh , Bioinformatics 2018, 32, 3246. 10.1093/bioinformatics/btw412 PMC5079479 27378296

[62] L. T. Nguyen , H. A. Schmidt , A. Von Haeseler , B. Q. Minh , Mol. Biol. Evol. 2015, 32, 268. 25371430 10.1093/molbev/msu300 PMC4271533

[63] S. Kalyaanamoorthy , B. Q. Minh , T. K. F. Wong , A. von Haeseler , L. S. Jermiin , Nat. Methods 2017, 14, 587. 28481363 10.1038/nmeth.4285 PMC5453245

[64] M. Tarailo‐Graovac , N. Chen , Curr Protoc Bioinformatics 2009, 1. 10.1002/0471250953.bi0410s25 19274634

[65] M. Kimura , J. Mol. Evol. 1980, 16, 111. 7463489 10.1007/BF01731581

[66] S. Afzal , S. Wilkening , C. von Kalle , M. Schmidt , R. Fronza , Mol. Ther. Nucleic Acids 2017, 6, 133. 28325279 10.1016/j.omtn.2016.12.001 PMC5363413

[67] H. Jiang , R. Lei , S.‐W. Ding , S. Zhu , BMC Bioinformatics 2014, 15, 182. 24925680 10.1186/1471-2105-15-182 PMC4074385

[68] I. Querques , A. Mades , C. Zuliani , C. Miskey , M. Alb , E. Grueso , M. Machwirth , T. Rausch , H. Einsele , Z. Ivics , M. Hudecek , O. Barabas , Nat. Biotechnol. 2019, 37, 1502. 31685959 10.1038/s41587-019-0291-z PMC6894935

