# Supplementary material for: A Naturally Active Spy Transposon Discovered from the Insect Genome of Colletes gigas as a Promising Novel Gene Transfer Tool
Source: Adv Sci (Weinh). 2024 May 22;11(29):2400969. doi: 10.1002/advs.202400969 (PMC11304231; doi:10.1002/advs.202400969)
Supplement: Supplementary file 1 — Supporting Information [file ADVS-11-2400969-s001.pdf]

ADVANCED SCIENCE
Open Access

Supporting Information

for *Adv. Sci.*, DOI 10.1002/advs.202400969

A Naturally Active *Spy* Transposon Discovered from the Insect Genome of *Colletes gigas* as a Promising Novel Gene Transfer Tool

*Mohamed Diaby, Han Wu, Bo Gao, Shasha Shi, Bingqing Wang, Saisai Wang, Yali Wang, Zherui Wu, Cai Chen, Xiaoyan Wang and Chengyi Song**

Figure S1

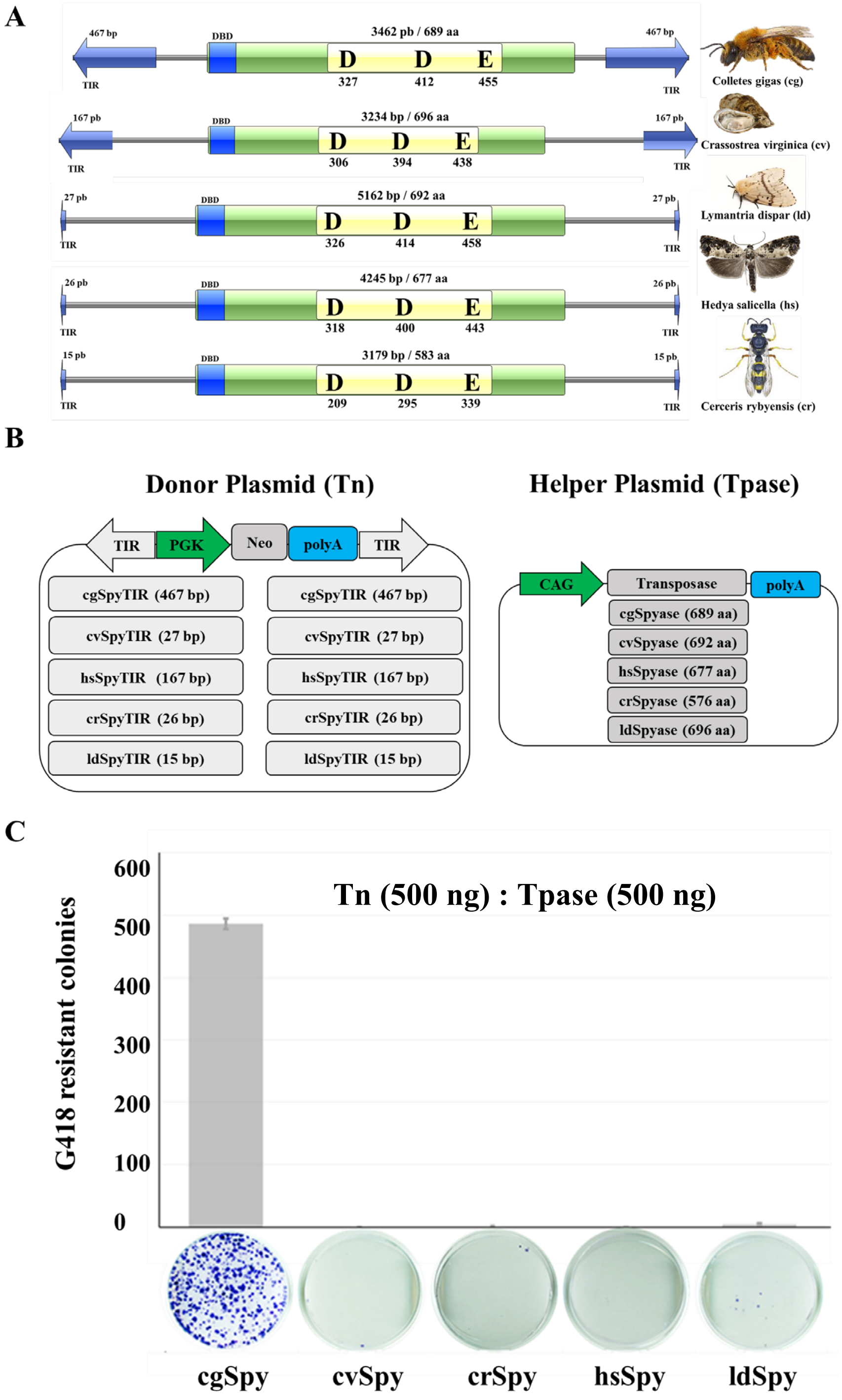

Figure S1. Transposition Activities of Spy Elements in Human Cells. (A) The structural and functional components of representative Spy transposons from *Colletes gigas*, *Crassostrea virginica*, *Hedya salicella Cerceris rybyensis*, and *Lymantria dispar* are depicted. The elements contain a single gene that encodes the transposase (green rectangle). The blue arrows represent TIRs, the blue rectangle represents the DNA-

binding domain (DBD), and the yellow rectangle represents the catalytic domain (DDE). (B) Donor and helper plasmids used in human cells. Donor plasmids: the white arrows represent transposon TIRs including cgSpyTIR, cvSpyTIR, hsSpyTIR, crSpyTIR, and ldSpyTIR; PGK, PGK promoter; Neo, neomycin resistance gene. Helper plasmids: CAG, CAG promoter; transposase, the transposase (cgSpyase, cvSpyase, hsSpyase, crSpyase, and ldSpyase) open reading frame (ORF). (C) Transposition activities of three Spy transposases (*cgSpy*, *cvSpy*, *hsSpy*, *crSpy*, and *ldSpy*) in HeLa cells co-transfected with different transposon DNA amounts (500 ng). Three replicates were performed for each group. The dishes represent G418-resistant cell colonies stained by Giemsa.

Figure S2

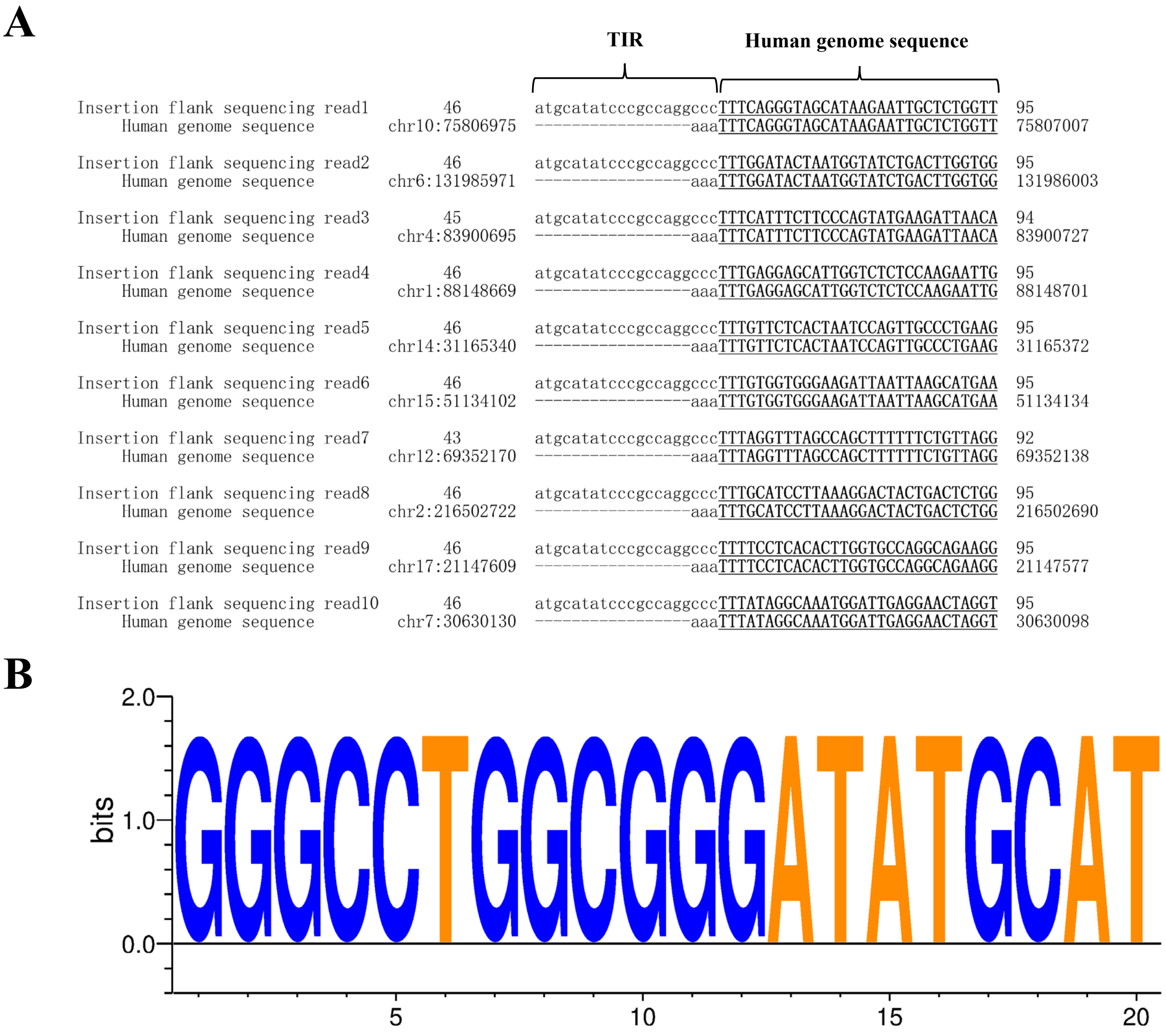

Figure S2. Insertion of *Spy* elements from *Colletes gigas* (*cgSpy*) into the human genome. (A) Each alignment depicts a single chromosomal insertion of *cgSpy*. Notably, these insertions consistently occur between AAA and TTT nucleotides. (B) This work generated a logo representing the first 20 bp sequences in the 5' TIR of *cgSpy* elements using the TBtools software (v1.0987663) from the GitHub repository (https://github.com/CJ-Chen/TBtools-II/releases).

Figure S3

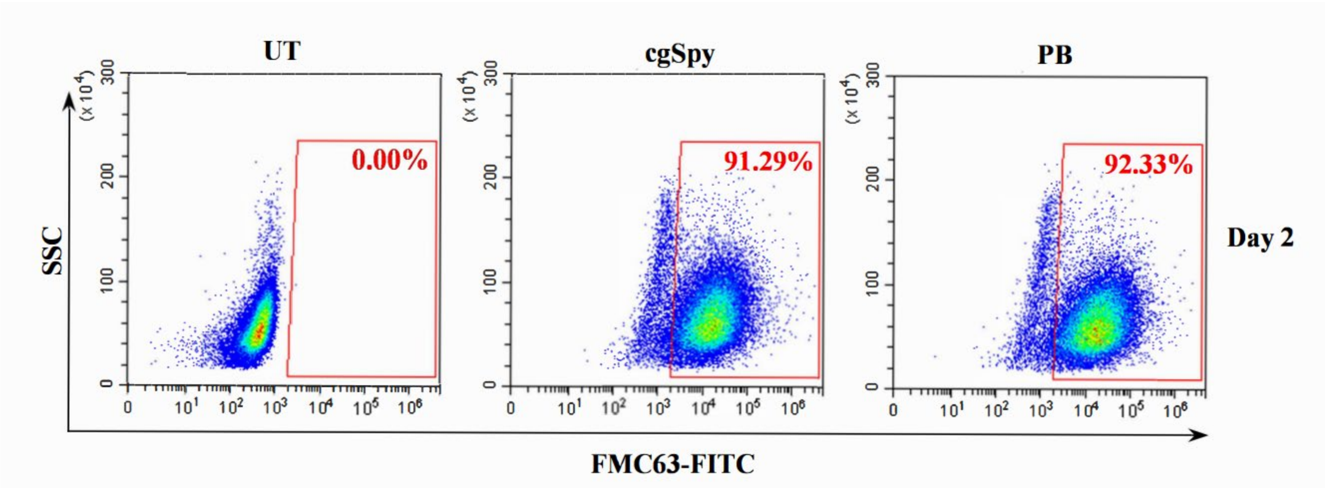

Figure S3. Determination of electrotransfection efficiencies of cgSpy- and PB-carried CD19-CAR in primary human T cells. One million activated primary human CD3+ T cells isolated from the PBMC of healthy donors were co-electroporated with 2μg of plasmid DNA loaded with transposon-loaded CD19-CAR and 2μg of transposase mRNA. After 2 days of transfection, flow cytometry analysis of CD19-CAR positive cells was conducted using FAM63-FITC antibody. Both the transfection rates of cgSpy and PB-carrying CD19-CAR exceeded 90%.

Table S1. Summary information of Spy transposon distributions across different phyla.

Phylum	Class	Order	Species	Tn name	Tn Length (bp)	TIR length (bp)	Number of ORF	ORF (aa)	Copy number (80% Id & 40% Cov.)	Copy number of intact Tns	Size(bp) Left/Right	TIR Identity %	Detected insertion motif
Arthropoda	Arachnida	Araneae	Oedothorax gibbosus	Oegi-Spy	1095	-	1	222	-	-	-	-	-
Arthropoda	Arachnida	Mesostigmata	Galendromus occidentalis	Gaoc-Spy-1	3958	4	1	463	4	-	41/41	0.88	AAA/TTT
Arthropoda	Arachnida	Mesostigmata	Galendromus occidentalis	Gaoc-Spy-2	2650	580	1	320	27	-	580/580	0.98	AAA/TTT
Arthropoda	Arachnida	Sarcoptiformes	Dermatophagoides farinae	Defa-Spy	4215	342	1	474	3	-	342/342	0.96	AAA/TTT
Arthropoda	Arachnida	Sarcoptiformes	Dermatophagoides pteronyssinus	Dept-Spy	1089	-	1	270	-	-	-	-	-
Arthropoda	Arachnida	Sarcoptiformes	Euroglyphus maynei	Euma-Spy	1046	-	1	246	-	-	-	-	-
Arthropoda	Arachnida	Scorpiones	Androctonus mauritanicus	Anma-Spy	3209	49	1	341	1	-	49/49	100	AAA/TTT
Arthropoda	Arachnida	Trombidiformes	Tetranychus urticae	Teur-Spy	1122	-	1	340	-	-	-	-	-
Arthropoda	Insecta	Coleoptera	Agrilus planipennis	Agpl-Spy	2691	94	1	690	2	1	94/94	0.968	AAA/TTT
Arthropoda	Insecta	Coleoptera	Cantharis rustica	Caru-Spy	3420	143	1	353	16	-	143/143	0.95	AAA/TTT
Arthropoda	Insecta	Coleoptera	Protaetia brevitarsis	Prbr-Spy	3525	441	1	311	33	-	441/441	0.945	AAA/TTT
Arthropoda	Insecta	Coleoptera	Diabrotica virgifera	Divi-Spy	1104	-	1	308	-	-	-	-	-
Arthropoda	Insecta	Coleoptera	Listronotus bonariensis	Libo-Spy	1061	-	1	296	-	-	-	-	-
Arthropoda	Insecta	Coleoptera	Leptinotarsa rubiginosa	Leru-Spy	1095	-	1	263	-	-	-	-	-
Arthropoda	Insecta	Coleoptera	Coccinella septempunctata	Cose-Spy	1022	-	1	259	-	-	-	-	-
Arthropoda	Insecta	Coleoptera	Pachyrhynchus sulphureomaculatus	Pasu-Spy	1003	-	1	231	-	-	-	-	-
Arthropoda	Insecta	Diptera	Dasypogon diadema	Dadi-Spy	2824	205	1	679	3	1	205/205	0.995	AAA/TTT
Arthropoda	Insecta	Diptera	Lordiphosa mommai	Lomo-Spy	2061	35	1	493	6	-	35/35	100	AAA/TTT
Arthropoda	Insecta	Diptera	Chymomyza costata	Chco-Spy	2808	312	1	457	19	-	312/312	0.99	AAA/TTT
Arthropoda	Insecta	Diptera	Lordiphosa stackelbergi	Lost-Spy	1092	-	1	304	-	-	-	-	-
Arthropoda	Insecta	Diptera	Lordiphosa clarofinis	Locl-Spy	1095	-	1	288	-	-	-	-	-
Arthropoda	Insecta	Diptera	Eutreta diana	Eudi-Spy	1049	-	1	247	-	-	-	-	-
Arthropoda	Insecta	Hemiptera	Hypogeococcus pungens	Hypu-Spy	3235	24	1	753	1	1	24/24	0.833	AAA/TTT
Arthropoda	Insecta	Hemiptera	Rhodnius prolixus	Rhpr-Spy	1667	152	1	320	2	-	152/152	100	AAA/TTT
Arthropoda	Insecta	Hemiptera	Trionymus perrisii	Trpe-Spy	3960	40	1	796	1	1	40/40	0.975	AAA/TTT
Arthropoda	Insecta	Hemiptera	Bemisia tabaci	Beta-Spy	1092	-	1	267	-	-	-	-	-
Arthropoda	Insecta	Hymenoptera	Adelognathus	AdAd-Spy	1725	133	1	304	4	-	133/133	100	AAA/TTT
Arthropoda	Insecta	Hymenoptera	Anastatus disparis	Andi-Spy	3961	220	1	446	7	-	220/220	100	AAA/TTT
Arthropoda	Insecta	Hymenoptera	Andrena haemorrhoa	Anha-Spy	3450	234	1	362	1	-	243/234	100	AAA/TTT
Arthropoda	Insecta	Hymenoptera	Andricus curtor	Ancu-Spy	1107	-	1	293	-	-	-	-	-
Arthropoda	Insecta	Hymenoptera	Andricus quercuslanigera	Anqu-Spy-1	3568	53	1	419	147	-	53/53	100	AAA/TTT
Arthropoda	Insecta	Hymenoptera	Andricus quercuslanigera	Anqu-Spy-2	1098	-	1	320	-	-	-	-	-
Arthropoda	Insecta	Hymenoptera	Aphaenogaster ashmeadi	Apas-Spy	3243	64	1	689	1	1	64/64	100	AAA/TTT
Arthropoda	Insecta	Hymenoptera	Aphaenogaster fulva	Apfu-Spy	1107	-	1	323	-	-	-	-	-
Arthropoda	Insecta	Hymenoptera	Aphaenogaster miamiana	Apmi-Spy	2574	20	1	204	1	-	20/20	100	AAA/TTT
Arthropoda	Insecta	Hymenoptera	Apocrypta bakeri	Apba-Spy	2945	365	1	309	18	-	365/356	0.948	AAA/TTT
Arthropoda	Insecta	Hymenoptera	Camponotus floridanus	Cafl-Spy	2872	65	1	688	4	3	65/65	0.984	AAA/TTT
Arthropoda	Insecta	Hymenoptera	Cerceris rybyensis	Cery-Spy-1	2921	49	1	768	4	2	49/49	100	AAA/TTT
Arthropoda	Insecta	Hymenoptera	Cerceris rybyensis	Cery-Spy-2	3234	167	1	696	8	7	167/167	0.929	AAA/TTT
Arthropoda	Insecta	Hymenoptera	Cerceris rybyensis	Cery-Spy-3	4507	614	1	768	130	1	614/614	100	AAA/TTT
Arthropoda	Insecta	Hymenoptera	Chilo suppressalis	Chsu-Spy	3350	401	1	702	1	1	401/401	0.937	AAA/TTT
Arthropoda	Insecta	Hymenoptera	Colletes gigas	Cogi-Spy	3462	467	1	689	14	14	467/467	0.99	AAA/TTT
Arthropoda	Insecta	Hymenoptera	Copidosoma floridanum	Cofl-Spy	1098	-	1	263	-	-	-	-	-
Arthropoda	Insecta	Hymenoptera	Cotesia chilonis	Coch-Spy	3501	448	1	624	3		448/448	0.984	AAA/TTT
Arthropoda	Insecta	Hymenoptera	Cotesia typhae	Coty-Spy	2384	21	1	461	8	-	21/21	0.761	AAA/TTT
Arthropoda	Insecta	Hymenoptera	Cotesia vestalis	Cove-Spy	5228	157	1	665	1	1	157/157	0.993	AAA/TTT
Arthropoda	Insecta	Hymenoptera	Crematogaster levior	Crle-Spy	1499	16	1	226	9	-	16/16	100	AAA/TTT
Arthropoda	Insecta	Hymenoptera	Cyphomyrmex costatus	Cyco-Spy	3461	352	1	690	1	1	352/352	0.97	AAA/TTT
Arthropoda	Insecta	Hymenoptera	Diachasma alloeum	Dial-Spy	4379	257	1	740	3	2	257/257	0.97	AAA/TTT
Arthropoda	Insecta	Hymenoptera	Ectemnius continuus	Ecco-Spy	3998	570	1	743	18	2	570/570	0.98	AAA/TTT
Arthropoda	Insecta	Hymenoptera	Eupelmus annulatus	Euan-Spy	5910	371	1	762	1	1	371/371	0.959	AAA/TTT
Arthropoda	Insecta	Hymenoptera	Eupelmus urozonus	Euur-Spy	1098	-	1	263	-	-	-	-	-
Arthropoda	Insecta	Hymenoptera	Eurytoma adleriae	Euad-Spy	2693	38	1	675	1	1	38/38	0.97	AAA/TTT
Arthropoda	Insecta	Hymenoptera	Eurytoma brunniventris	Eubr-Spy	2701	17	1	703	1	1	17/17	0.82	AAA/TTT
Arthropoda	Insecta	Hymenoptera	Euura lappo	Eula-Spy	3847	454	1	584	1	1	454/454	0.988	AAA/TTT
Arthropoda	Insecta	Hymenoptera	Euura saliciscinereae	Eusa-Spy	3351	41	1	625	1	1	41/41	100	AAA/TTT
Arthropoda	Insecta	Hymenoptera	Exoneura robusta	Exro-Spy	1095	-	1	262	-	-	-	-	-
Arthropoda	Insecta	Hymenoptera	Formica aquilonia	Foaq-Spy	1092	-	1	220	-	-	-	-	-
Arthropoda	Insecta	Hymenoptera	Formica exsecta	Foex-Spy	2443	25	1	309	8	-	25/25	100	AAA/TTT
Arthropoda	Insecta	Hymenoptera	Formica selysi	Fose-Spy-1	3598	310	1	679	50	50	310/310	0.98	AAA/TTT
Arthropoda	Insecta	Hymenoptera	Formica selysi	Fose-Spy-2	3874	123	1	562	2	1	123/123	100	AAA/TTT
Arthropoda	Insecta	Hymenoptera	Formica selysi	Fose-Spy-3	5158	19	1	311	15	-	19/19	0.84	AAA/TTT
Arthropoda	Insecta	Hymenoptera	Formica selysi	Fose-Spy-4	3596	309	1	679	50	38	309/309	0.987	AAA/TTT
Arthropoda	Insecta	Hymenoptera	Gonatopus flavifemur	Gofl-Spy	5643	110	1	354	4	-	110/110	0.98	AAA/TTT
Arthropoda	Insecta	Hymenoptera	Lasioglossum albipes	Laal-Spy	1098	-	1	320	-	-	-	-	-
Arthropoda	Insecta	Hymenoptera	Lasius niger	Lani-Spy	2890	62	1	686	1	1	62/62	100	AAA/TTT
Arthropoda	Insecta	Hymenoptera	Leptopilina boulardi	Lebo-Spy	5512	22	1	688	2	1	22/22	100	AAA/TTT
Arthropoda	Insecta	Hymenoptera	Leptopilina heterotoma	Lehe-Spy	3191	124	1	686	4	3	124/124	0.976	AAA/TTT
Arthropoda	Insecta	Hymenoptera	Macrocentrus cingulum	Maci-Spy	1098	-	1	320	-	-	-	-	-
Arthropoda	Insecta	Hymenoptera	Magicicada septendecim	Mase-Spy	1098	-	1	327	-	-	-	-	-
Arthropoda	Insecta	Hymenoptera	Magicicada septendecula	Mase-Spy	1095	-	1	326	-	-	-	-	-
Arthropoda	Insecta	Hymenoptera	Megalopta genalis	Mege-Spy	1095	-	1	306	-	-	-	-	-
Arthropoda	Insecta	Hymenoptera	Megastigmus dorsalis	Medo-Spy	1095	-	1	334	-	-	-	-	-
Arthropoda	Insecta	Hymenoptera	Megastigmus stigmatizans	Mest-Spy	3755	686	1	250	6	-	686/686	0.976	AAA/TTT
Arthropoda	Insecta	Hymenoptera	Microplitis demolitor	Mide-Spy	1095	-	1	319	-	-	-	-	-

Arthropoda	Insecta	Hymenoptera	Monomorium pharaonis	Moph-Spy	3710	45	1	709	10	1	45/45	0.93	AAA/TTT
Arthropoda	Insecta	Hymenoptera	Nasonia giraulti	Nagi-Spy	1095	-	1	267	-	-	-	-	-
Arthropoda	Insecta	Hymenoptera	Nasonia vitripennis	Navi-Spy	1101	-	1	269	-	-	-	-	-
Arthropoda	Insecta	Hymenoptera	Neodiprion lecontei	Nele-Spy	3753	159	1	355	14	-	159/159	0.987	AAA/TTT
Arthropoda	Insecta	Hymenoptera	Neodiprion pinetum	Nepi-Spy	2379	21	1	568	2	2	21/21	0.857	AAA/TTT
Arthropoda	Insecta	Hymenoptera	Neuroterus quercusbaccarum	Nequ-Spy	1098	-	1	320	-	-	-	-	-
Arthropoda	Insecta	Hymenoptera	Nomada fabriciana	Nofa-Spy	1076	-	1	262	-	-	-	-	-
Arthropoda	Insecta	Hymenoptera	Nysson spinosus	Nysp-Spy	4150	57	1	912	13	1	57/57	100	AAA/TTT
Arthropoda	Insecta	Hymenoptera	Odontomachus brunneus	Odbr-Spy	1058	-	1	251	-	-	-	-	-
Arthropoda	Insecta	Hymenoptera	Ooceraea biroi	Oobi-Spy	1521	11	1	447	4	-	11/11	0.9	AAA/TTT
Arthropoda	Insecta	Hymenoptera	Ormyrus nitidulus	Orni-Spy	4106	374	1	280	1	-	374/374	0.92	AAA/TTT
Arthropoda	Insecta	Hymenoptera	Ormyrus pomaceus	Orpo-Spy	1098	-	1	312	-	-	-	-	-
Arthropoda	Insecta	Hymenoptera	Osmia bicornis	Osbi-Spy	4726	28	1	417	7	-	28/28	100	AAA/TTT
Arthropoda	Insecta	Hymenoptera	Osmia lignaria	Osli-Spy	1092	-	1	289	-	-	-	-	-
Arthropoda	Insecta	Hymenoptera	Phanerotoma	PhPh-Spy	2958	88	1	698	2	1	88/88	100	AAA/TTT
Arthropoda	Insecta	Hymenoptera	Polistes dominula	Podo-Spy	3197	23	1	457	19	-	23/23	0.615	AAA/TTT
Arthropoda	Insecta	Hymenoptera	Pseudomyrmex cubaensis	Pscu-Spy	1098	-	1	320	-	-	-	-	-
Arthropoda	Insecta	Hymenoptera	Pseudomyrmex gracilis	Psgr-Spy	1098	-	1	320	-	-	-	-	-
Arthropoda	Insecta	Hymenoptera	Solenopsis fugax	Sofu-Spy	1092	-	1	318	-	-	-	-	-
Arthropoda	Insecta	Hymenoptera	Solenopsis invicta	Soin-Spy	1165	11	1	305	28	-	11/11	0.727	AAA/TTT
Arthropoda	Insecta	Hymenoptera	Synergus gifuensis	Sygi-Spy	4064	11	1	348	3	-	11/11	0.9	AAA/TTT
Arthropoda	Insecta	Hymenoptera	Synergus itoensis	Syit-Spy	4711	244	1	694	2	1	244/244	0.95	AAA/TTT
Arthropoda	Insecta	Hymenoptera	Synergus japonicus	Syja-Spy	4041	80	1	302	36	-	80/80	0.987	AAA/TTT
Arthropoda	Insecta	Hymenoptera	Temnothorax curvispinosus	Tecu-Spy	1098	-	1	320	-	-	-	-	-
Arthropoda	Insecta	Hymenoptera	Temnothorax longispinosus	Telo-Spy	3455	193	1	690	1	1	193/193	0.989	AAA/TTT
Arthropoda	Insecta	Hymenoptera	Tetramorium parvispinum	Tepa-Spy	2167	34	1	550	1	1	34/34	0.94	AAA/TTT
Arthropoda	Insecta	Hymenoptera	Trachymyrmex cornetzi	Trco-Spy	3723	492	1	320	8	-	492/492	0.959	AAA/TTT
Arthropoda	Insecta	Hymenoptera	Trichogramma evanescens	Trev-Spy	1092	-	1	261	-	-	-	-	-
Arthropoda	Insecta	Hymenoptera	Trichogramma pretiosum	Trpr-Spy	1073	-	1	213	-	-	-	-	-
Arthropoda	Insecta	Hymenoptera	Venturia canescens	Veca-Spy	4214	427	1	706	2	2	427/427	0.943	AAA/TTT
Arthropoda	Insecta	Hymenoptera	Vollenhovia emeryi	Voem-Spy	2513	34	1	691	1	1	34/34	0.914	AAA/TTT
Arthropoda	Insecta	Hymenoptera	Wasmannia auropunctata	Waau-Spy	1098	-	1	263	-	-	-	-	-
Arthropoda	Insecta	Lepidoptera	Aeromachus stigmata	Aest-Spy	3418	17	1	668	1	1	17/17	0.941	AAA/TTT
Arthropoda	Insecta	Lepidoptera	Agrotis ipsilon	Agip-Spy	4820	214	1	509	18	1	214/214	100	AAA/TTT
Arthropoda	Insecta	Lepidoptera	Aguna metophis	Agme-Spy	1334	5	1	414	1	-	5/5	100	AAA/TTT
Arthropoda	Insecta	Lepidoptera	Amphipyra tragopoginis	Amtr-Spy	4488	12	1	517	5	1	12/12	100	AAA/TTT
Arthropoda	Insecta	Lepidoptera	Ampittia dioscorides	Amdi-Spy	3136	7	1	647	1	1	7/7	100	AAA/TTT
Arthropoda	Insecta	Lepidoptera	Anthocharis cardamine	Anca-Spy	1049	-	1	304	-	-	-	-	-
Arthropoda	Insecta	Lepidoptera	Anthocharis cardamines	Anca-Spy	3532	356	1	715	3	2	356/356	100	AAA/TTT
Arthropoda	Insecta	Lepidoptera	Apamea monoglypha	Apmo-Spy	3542	443	1	700	47	1	443/443	100	AAA/TTT
Arthropoda	Insecta	Lepidoptera	Apodemia chisosensis	Apch-Spy	3340	108	1	700	1	1	108/108	0.953	AAA/TTT
Arthropoda	Insecta	Lepidoptera	Apotomis turbidana	Aptu-Spy	4396	299	1	674	2	1	299/299	0.973	AAA/TTT
Arthropoda	Insecta	Lepidoptera	Apyrrothrix maculosa	Apma-Spy	2568	40	1	712	1	1	40/40	0.8	AAA/TTT
Arthropoda	Insecta	Lepidoptera	Apyrrothrix mulleri	Apmu-Spy	1250	6	1	320	1	-	6/6	100	AAA/TTT
Arthropoda	Insecta	Lepidoptera	Archon apollinus	Arap-Spy	2790	43	1	715	1	1	43/43	0.837	AAA/TTT
Arthropoda	Insecta	Lepidoptera	Autochton itylus	Auit-Spy	1386	18	1	313	1	-	18/18	0.888	AAA/TTT
Arthropoda	Insecta	Lepidoptera	Bibasis iluska	Biil-Spy	2795	96	1	706	1	1	96/96	0.968	AAA/TTT
Arthropoda	Insecta	Lepidoptera	Boloria selene	Bose-Spy	3411	440	1	693	3	1	440/440	0.986	AAA/TTT
Arthropoda	Insecta	Lepidoptera	Cecropterus bathyllus	Ceba-Spy	1098	-	1	320	-	-	-	-	-
Arthropoda	Insecta	Lepidoptera	Cydia pomonella	Cypo-Spy	2751	48	1	453	3	-	48/48	0.979	AAA/TTT
Arthropoda	Insecta	Lepidoptera	Cydia splendana	Cysp-Spy	2896	151	1	661	12	8	151/151	0.993	AAA/TTT
Arthropoda	Insecta	Lepidoptera	Deilephila porcellus	Depo-Spy	1086	-	1	302	-	-	-	-	-
Arthropoda	Insecta	Lepidoptera	Dryas iulia	Driu-Spy	3333	362	1	697	10	1	362/362	100	AAA/TTT
Arthropoda	Insecta	Lepidoptera	Ectropis grisescens	Ecgr-Spy	1043	-	1	280	-	-	-	-	-
Arthropoda	Insecta	Lepidoptera	Emesis diogenia	Emdi-Spy	3398	35	1	648	1	1	35/35	0.94	AAA/TTT
Arthropoda	Insecta	Lepidoptera	Emesis melancholica	Emme-Spy	1092	-	1	304	-	-	-	-	-
Arthropoda	Insecta	Lepidoptera	Ernsta delagoae	Erde-Spy	3038	15	1	560	1	1	15/15	0.93	AAA/TTT
Arthropoda	Insecta	Lepidoptera	Eumeta japonica	Euja-Spy	4392	249	1	676	4	1	249/249	0.98	AAA/TTT
Arthropoda	Insecta	Lepidoptera	Glaucopsyche alexis	Glal-Spy	5576	22	1	552	5	1	22/22	0.77	AAA/TTT
Arthropoda	Insecta	Lepidoptera	Hasora badra	Haba-Spy	2549	32	1	716	1	1	32/32	0.812	AAA/TTT
Arthropoda	Insecta	Lepidoptera	Hecatera dysodea	Hedy-Spy	3554	490	1	699	4	2	490/490	0.98	AAA/TTT
Arthropoda	Insecta	Lepidoptera	Hedya salicella	Hesa-Spy	4245	26	1	677	12	11	26/26	93	AAA/TTT
Arthropoda	Insecta	Lepidoptera	Helicoverpa armigera	Hear-Spy	4775	12	1	665	19	16	12/12	100	AAA/TTT
Arthropoda	Insecta	Lepidoptera	Hemaris fuciformis	Hefu-Spy	3674	435	1	691	3	2	435/435	0.98	AAA/TTT
Arthropoda	Insecta	Lepidoptera	Hyles vespertilio	Hyve-Spy	3374	455	1	689	4	3	455/455	0.99	AAA/TTT
Arthropoda	Insecta	Lepidoptera	Junonia coenia	Juco-Spy	3055	13	1	536	5	1	13/13	0.92	AAA/TTT
Arthropoda	Insecta	Lepidoptera	Junonia erete	Juer-Spy	1095	-	1	301	-	-	-	-	-
Arthropoda	Insecta	Lepidoptera	Junonia evarete	Juev-Spy	3231	191	1	593	2	1	191/191	0.968	AAA/TTT
Arthropoda	Insecta	Lepidoptera	Junonia genove	Juge-Spy	1098	-	1	294	-	-	-	-	-
Arthropoda	Insecta	Lepidoptera	Junonia grisea	Jugr-Spy	1095	-	1	301	-	-	-	-	-
Arthropoda	Insecta	Lepidoptera	Junonia neildi	June-Spy	1098	-	1	294	-	-	-	-	-
Arthropoda	Insecta	Lepidoptera	Junonia oenone	Juoe-Spy	1095	-	1	311	-	-	-	-	-
Arthropoda	Insecta	Lepidoptera	Junonia vestina	Juve-Spy	1101	-	1	292	-	-	-	-	-
Arthropoda	Insecta	Lepidoptera	Junonia villida	Juvi-Spy	1095	-	1	301	-	-	-	-	-
Arthropoda	Insecta	Lepidoptera	Junonia zonalis	Juzo-Spy	1107	-	1	305	-	-	-	-	-
Arthropoda	Insecta	Lepidoptera	Katreus johnstonii	Kajo-Spy	1098	-	1	320	-	-	-	-	-
Arthropoda	Insecta	Lepidoptera	Leucinodes orbonalis	Leor-Spy	3343	32	1	544	5	3	32/32	100	AAA/TTT
Arthropoda	Insecta	Lepidoptera	Lymantria dispar	Lydi-Spy	3182	15	1	576	851	402	15/15	100	AAA/TTT
Arthropoda	Insecta	Lepidoptera	Lymantria monacha	Lymo-Spy	3179	15	1	583	52	9	15/15	100	AAA/TTT
Arthropoda	Insecta	Lepidoptera	Mellicta athalia	Meat-Spy-1	1095	-	1	319	-	-	-	-	-
Arthropoda	Insecta	Lepidoptera	Mellicta athalia	Meat-Spy-2	3724	691	1	676	1	1	691/691	0.99	AAA/TTT
Arthropoda	Insecta	Lepidoptera	Mellicta athalia	Meat-Spy-3	2542	14	1	676	1	1	14/14	0.857	AAA/TTT

Arthropoda	Insecta	Lepidoptera	Metardaris cosinga	Meco-Spy	1061	-	1	308	-	-	-	-	-
Arthropoda	Insecta	Lepidoptera	Muschampia kuenlunus	Muku-Spy	1092	-	1	310	-	-	-	-	-
Arthropoda	Insecta	Lepidoptera	Mysoria ambigua	Myam-Spy	1061	-	1	308	-	-	-	-	-
Arthropoda	Insecta	Lepidoptera	Mysoria barcastus	Myba-Spy	1061	-	1	308	-	-	-	-	-
Arthropoda	Insecta	Lepidoptera	Mysoria catomelaena	Myca-Spy	1076	-	1	313	-	-	-	-	-
Arthropoda	Insecta	Lepidoptera	Mythimna ferrago	Myfe-Spy	2084	12	1	336	3	-	12/12	0.916	AAA/TTT
Arthropoda	Insecta	Lepidoptera	Notocelia uddmanniana	Noud-Spy	4177	68	1	558	3	1	68/68	0.95	AAA/TTT
Arthropoda	Insecta	Lepidoptera	Pammene fasciana	Pafa-Spy	2983	307	1	441	1	-	307/307	0.987	AAA/TTT
Arthropoda	Insecta	Lepidoptera	Papilio machaon	Pama-Spy	3998	18	1	458	2	-	18/18	0.83	AAA/TTT
Arthropoda	Insecta	Lepidoptera	Papilio zelicaon	Paze-Spy	2473	47	1	712	1	1	47/47	100	AAA/TTT
Arthropoda	Insecta	Lepidoptera	Polygonus leo	Pole-Spy	3326	38	1	689	1	1	38/38	0.97	AAA/TTT
Arthropoda	Insecta	Lepidoptera	Toxidia parvulus	Topa-Spy	2651	55	1	702	1	1	55/55	100	AAA/TTT
Arthropoda	Insecta	Lepidoptera	Zeuzera pyrina	Zepy-Spy	1255	15	1	316	1590	-	15/15	0.93	AAA/TTT
Arthropoda	Insecta	Neuroptera	Chrysoperla carnea	Chca-Spy	2724	94	1	691	1	1	94/94	0.957	AAA/TTT
Arthropoda	Insecta	Orthoptera	Apteronemobius asahinai	Apas-Spy	1095	-	1	203	-	-	-	-	-
Arthropoda	Insecta	Orthoptera	Vandiemenella viatica	Vavi-Spy	1022	-	1	211	-	-	-	-	-
Arthropoda	Insecta	Siphonaptera	Ctenocephalides felis	Ctfe-Spy	3927	562	1	678	20	12	562/562	0.99	AAA/TTT
Arthropoda	Insecta	Strepsiptera	Mengenilla moldrzyki	Memo-Spy	2643	8	1	653	2	1	8/8	100	AAA/TTT
Cnidaria	Anthozoa	Actiniaria	Actinia tenebrosa	Acte-Spy	4115	-	1	594	-	-	-	-	-
Cnidaria	Hydrozoa	Anthoathecata	Hydra vulgaris	Hyvu-Spy-1	3517	21	1	963	102	2	21/21	0.809	AAA/TTT
Cnidaria	Hydrozoa	Anthoathecata	Hydra vulgaris	Hyvu-Spy-2	2612	29	1	241	95	-	29/29	0.666	AAA/TTT
Mollusca	Bivalvia	Ostreida	Crassostrea gigas	Crgi-Spy	5236	37	1	685	4	1	37/37	0.972	AAA/TTT
Mollusca	Bivalvia	Ostreida	Crassostrea virginica	Crvi-Spy	5162	27	1	455	10	-	27/27	96	AAA/TTT
Mollusca	Bivalvia	Pectinida	Mizuhopecten yessoensis	Miye-Spy	4768	-	1	284	-	-	-	-	-
Mollusca	Bivalvia	Pectinida	Pecten maximus	Pema-Spy	7586	-	1	372	-	-	-	-	-

The intact copy number of transposons refers to the count of complete transposons that possess two TIRs and intact transposases. These intact transposases comprise two DBD motifs and a DDE catalytic domain. Transposase identity was determined by comparing all intact Spy transposase sequences within a specific genome. TIR identity was determined by comparing both the 5' and 3' TIRs in each identified element

Table S2. Summary information of Spy transposon used for the evolutionary dynamic analysis

Species	Length (bp)	TIR length (bp)	ORF (aa)	Copy number (80% Id & 40% Cov.)	Copy number of intact Tns	Size (bp) L/R	Tn Identity %	Tpase Identity %	TIR (L/R) Identity %
Cerceris rybyensis (Spy2)	3234	167	696	8	7	167/167	99	99	93
Colletes gigas	3462	467	689	14	14	467/467	79	85	99
Crassostrea virginica	5162	27	692	10	8	27/27	97	99	96
Ctenocephalides felis	3927	562	678	20	12	562/562	77	44	99
Cydia splendana	2896	151	661	12	8	151/151	72	89	99
Formica selysi (Spy1)	3598	310	679	50	50	310/310	74	58	98
Hedya salicella	4245	26	677	12	11	26/26	99	99	93
Helicoverpa armigera	4775	12	665	45	28	12/12	99	72	100
Lymantria dispar	3182	15	576	851	402	15/15	98	98	100
Lymantria monacha	3179	15	583	52	9	15/15	98	49	100

Table S3. Primers used in this study

Primers group	Primer name	Sequence (5’-3’)	Function
Primers used for cgSpy, cvSpy, hsSpy TIR and transposase clones	cgSpy-TIR-F-1	GCTAGCAAAGGGCCTGGCGGGATAT	
	cgSpy-TIR-F-2	CCTAGGGCAGCCTCCTTGCGCTCCG	
	cgSpy-TIR-R-1	GAATTCAAAGGGCCTGGCGGGATAT	
	cgSpy-TIR-R-2	ACGCGTGCAGCCTCCTTGCGCTCCG	
	cgSpy-ORF-F	ATGTCCAATGCTGAGAAATGTGCGT	
	cgSpy-ORF-R	TTAATCATCGATATCGCTGTTCTCA	
	cvSpy-TIR-F	AAAGAGCCAAGCCCAGGAAGGGTCA	
	cvSpy-TIR-R	AAAGAGCCAAGCCCACGAAGGGTCA	
	cvSpy-TIR-UTR-F	GCTAGCAAAGAGCCAAGCCCAGGAAGGGTCAAAATCAGCA	
	cvSpy-TIR-UTR -R	CCGCGGAAAGAGCCAAGCCCACGAAGGGTCAAATCCGGCC	
	cvSpy-ORF-F	CCCGGGTAATACGACTCACTATAGG	
	cvSpy-ORF-R	TCATTCCTCCACCACTGATCCATCA	
	hsSpy-TIR-F	AAATCGGGATTAACAGTGTGAAAAA	
	hsSpy-TIR-R	AAATCAGGATTAACAGTTTGAAAAAT	

	hsSpy-TIR-UTR -F	GCTAGCAAATCGGGATTAACAGTGTGAAAAATTTGATTTT	
	hsSpy-TIR-UTR -R	CCGCGGAAATCAGGATTAACAGTTTGAAAAATCGAATTTG	
	crSpy-TIR -F	AAAGGACCCCGCACAAACCTTATGG	
	crSpy-TIR- R	CCATAAGATTTGTGCGGGGTCCTTT	
	crSpy-TIR-UTR-F	GCTAGCAAAGGACCCCGCACAAACCTTATGGATTTTGGAT	
	crSpy-TIR-UTR -R	ATCCAAAATCCATAAGATTTGTGCGGGGTCCTTTCCGCGG	
	ldSpy-TIR -F	AAAGGACAATGGGCATTGCGGCCCA	
	ldSpy-TIR- R	CATACATTTTTGCCCATTGTCCTTT	
	ldSpy-TIR-UTR-F	GCTAGCAAAGGACAATGGGCATTGCGGCCCATGAGCGGCC	
	ldSpy-TIR-UTR -R	CCGCGGAAAGGACAATGGGCAAAAATGTATGGCGCTTGTC	
	hsSpy-ORF-F	ATGGAGATGAGCGCCAATAAGTACC	
	hsSpy-ORF-R	ATCATCGTCAGAGTCAGACTGAATA	
Primers used for the cargo capacity of the cgSpy transposon	λ 1-F	AGGCATGCTGGGGAGAATTCGGATGGTGATGCCGAGAACT	
	λ 1-R	CGCAAGGAGGCTGCGAATTCCATTGCGTCGCTTTTTGCTGT	
	λ S1-F	TTTTTTGAACGAGGTTTAGAGCAAGCTTAGGCACTC	
	λ S1-R	TGTCTCAGTTTCCTGAAGCTTCCCGTCCAAGCCAGA	

	λ 2-F	GCATGCTGGGGAGAATTCGGATGGTGATGCCGAGAA	
	λ 2-R	CGCAAGGAGGCTGCGAATTCGCTGGCAATATGCGGGAGAT	
	λ S2-F	GAACGAGGTTTAGAGCAAGCTTATGGCCTTTAATGAGCCG	
	λ S2-R	TGTCTCAGTTTCCTGAAGCTTCCCGTCCAAGCCAGAGATGA	
	λ 3-F	AGGCATGCTGGGGAGAATTCGGATGGTGATGCCGAGAACT	
	λ 3-R	AGCGCAAGGAGGCTGCGAATTCCCCGTCCAAGCCAGAGAT	
	λ S4-F	TTTTGAACGAGGTTTAGAGCGGATGGTGATGCCGAGAACT	
	λ S4-R	GTCTCAGTTTCCTGAAGCTTCCCGTCCAAGCCAGAGATGA	
Primers used for insertion copy number detection of cgSpy	Neo-F	GCAACTAGAAGGCACAGTCG	
	Neo-R	GGCCGCTTTTCTGGATTCAT	
	Neo-probe	FAM-TGATCCCCTCAGAAGAACTCG-BHQ1	
	RPP30-F	AGATTTGGACCTGCGAGCG	
	RPP30-R	GAGCGGCTGTCTCCACAAGT	
	RPP30-probe	HEX-TTCTGACCTGAAGGCTCTGCGCG-BHQ1	
	CD19-probe	FAM-AAGCTGGAGATCACAGGTGG-BHQ1	
	CD19-F	AGCAACCTGGAGCAAGAAGA	

	CD19-R	CAGTTTCACCTCAGATCCGC	
Primers used for integration site analysis	cgSpy-ITR-F	TACATACATCTGCCGCCCATC	Positive identification
	cgSpy-ITR-R	TCCGCGTGCTACACCCATTT	Positive identification
	cgSpy-F1 bio	TTTTAGATGGGCGGCAGATG	Primer extension
	cgSpy-F2 bio	CGGGTTCAAAATTAAGGAAAG	1ST Nest PCR
	megaprimer	AAAATCAAACTAATCTGTTTGGGG	2ND Nest PCR
